# Precision Survival Estimation in Acute Myeloid Leukemia Using Evolutionary Learning-Derived microRNA Signature

**DOI:** 10.34133/csbj.0260

**Published:** 2026-11-11

**Authors:** Srinivasulu Yerukala Sathipati, David Agustriawan, Naga Sumanth Reddy Gopireddy, Apurva Popat, Luke Moat, Nikhila Aimalla, Manya Reddy Elugoti, Sai Akshay Kampa, Param Sharma, Shinn-Ying Ho, Rohit Sharma

**Affiliations:** ^1^ Center for Precision Medicine Research, Marshfield Clinic Research Institute, Sanford Health–Marshfield Clinic, Marshfield, WI, USA.; ^2^ Department of Informatics, Faculty of Engineering and Informatics, Universitas Multimedia Nusantara, Tangerang, Indonesia.; ^3^ Department of Internal Medicine, Guthrie Robert Packer Hospital, Sayre, PA, USA.; ^4^ Department of Cardiology, Sanford Health–Marshfield Clinic, Marshfield, WI, USA.; ^5^ Department of Internal Medicine, Division of Oncology and Hematology, College of Medicine, University of Nebraska Medical Center, Omaha, NE, USA.; ^6^ Edison Academy Magnet School, Edison, NJ, USA.; ^7^ Government Medical College, Nandyala, AP, India.; ^8^ Institute of Bioinformatics and Systems Biology, National Yang Ming Chiao Tung University, Hsinchu, Taiwan.; ^9^ Department of Biological Science and Technology, National Yang Ming Chiao Tung University, Hsinchu, Taiwan.; ^10^ Center for Intelligent Drug Systems and Smart Bio-devices (IDS2B), National Yang Ming Chiao Tung University, Hsinchu, Taiwan.; ^11^ College of Health Sciences, Kaohsiung Medical University, Kaohsiung, Taiwan.; ^12^ Department of Surgical Oncology, Sanford Health–Marshfield Clinic, Marshfield, WI, USA.

## Abstract

Acute myeloid leukemia (AML) remains associated with poor long-term survival despite advances in targeted therapies. MicroRNAs (miRNAs) regulate hematopoietic differentiation, apoptosis, and therapeutic resistance and may provide complementary markers of disease outcome. We applied the AML Survival Estimator, which integrates evolutionary feature selection with support vector regression, to expression profiles from The Cancer Genome Atlas acute myeloid leukemia cohort, comprising 354 miRNA features from 156 patients and identified a 28-miRNA candidate panel. Linked clinical records documented 96 deaths and 60 censored observations; Kaplan–Meier median overall survival was 19.23 months. The panel-based Cox model achieved Harrell concordance of 0.718, a 24-month time-dependent area under the curve of 0.852, and an integrated Brier score of 0.164 over 6 to 36 months. Concordance was 0.645 for an age/sex model and 0.730 for the combined model. Permutation tests, size-matched random-panel controls, early-outcome sensitivity analyses, and calibration assessment further characterized performance. Qualitative target and pathway annotations highlighted candidate links to leukemogenesis and apoptosis. These findings identify a compact miRNA panel for AML prognostic research and support independent evaluation alongside established clinical and molecular risk models.

## Graphical Abstract

**Figure:**
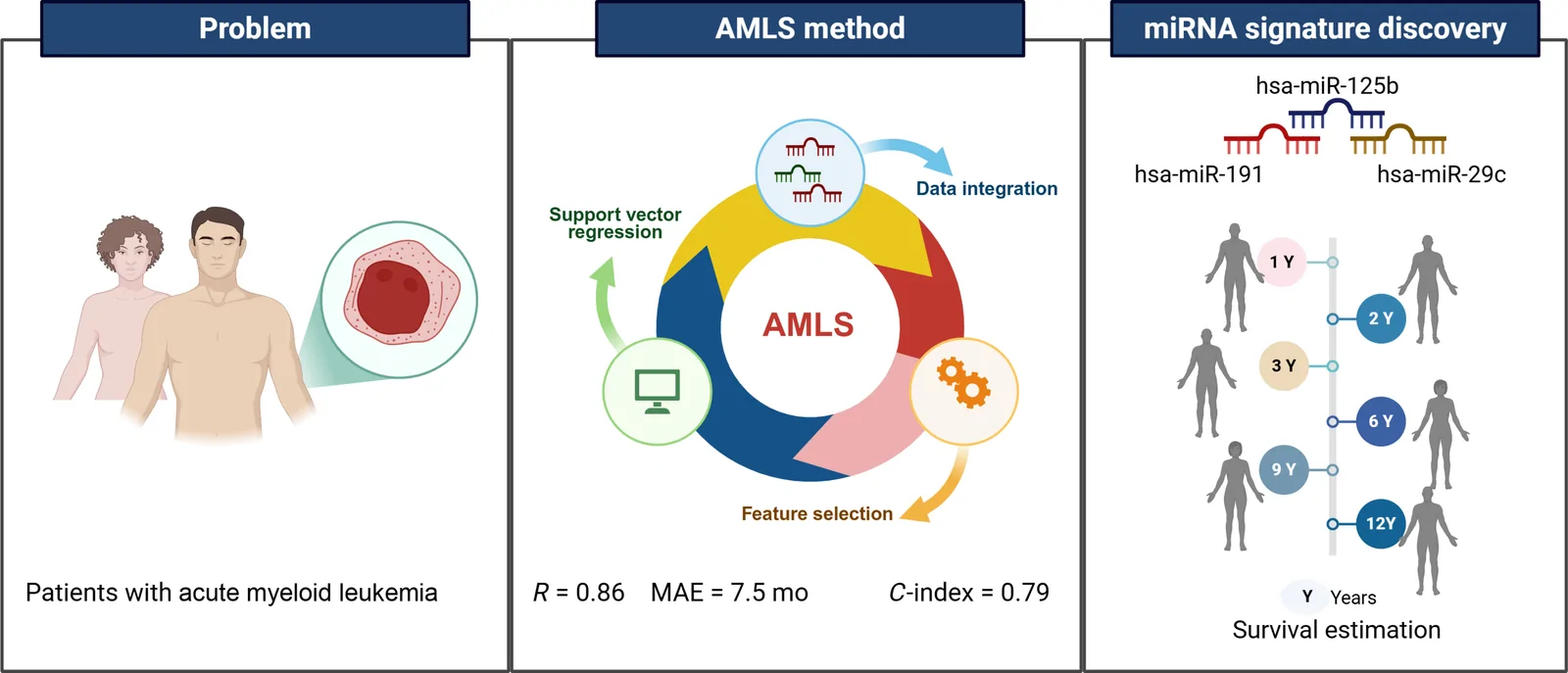

## Introduction

Acute myeloid leukemia (AML) is a genetically and clinically heterogeneous hematological malignancy characterized by impaired myeloid differentiation and uncontrolled proliferation of immature myeloid progenitors [1–3]. Despite substantial advances in molecular diagnostics and targeted therapeutics, AML remains associated with high mortality in adults, particularly among older patients and those with adverse-risk disease [1–4]. Recent therapeutic developments, including venetoclax-based regimens, FLT3 inhibitors, IDH1/2-targeted therapies, and emerging Menin inhibitors, have substantially expanded treatment options and improved remission rates in selected molecular subgroups [5–8]. Nevertheless, durable responses remain inconsistent, relapse rates remain high, and survival outcomes continue to vary markedly across patients with apparently similar clinicogenomic profiles [9,10]. These observations highlight the need for additional molecular biomarkers capable of refining prognostic assessment and supporting more individualized therapeutic decision-making.

Current AML risk stratification frameworks are primarily based on cytogenetic abnormalities and recurrent somatic mutations. The European LeukemiaNet (ELN-2022) classification has substantially improved clinical risk assessment through integration of cytogenetic findings with molecular alterations involving genes such as *NPM1*, *FLT3*, *CEBPA*, *RUNX1*, *ASXL1*, and *TP53* [1]. However, significant heterogeneity persists even within established risk groups, particularly among intermediate-risk patients, where clinical outcomes can vary dramatically despite shared genomic features [6,9]. Importantly, most current biomarkers primarily capture structural genomic alterations and may incompletely reflect the dynamic regulatory networks that govern disease progression, therapeutic resistance, stemness, and relapse biology. Consequently, increasing attention has shifted toward regulatory noncoding RNAs, particularly microRNAs (miRNAs), as potential orthogonal biomarkers capable of capturing these biologically relevant processes [11–13].

MicroRNAs are small noncoding RNAs, approximately 22 nucleotides in length, that regulate gene expression post-transcriptionally through translational repression or mRNA degradation [11,13]. They play essential roles in normal hematopoiesis, including stem-cell maintenance, lineage commitment, proliferation, apoptosis, immune regulation, and differentiation [12,14,15]. Dysregulation of miRNA expression has been implicated in numerous hematological malignancies and contributes directly to leukemogenesis through modulation of oncogenic and tumor suppressor pathways [12,16,17]. In AML, distinct miRNA signatures have been associated with cytogenetic and molecular subtypes, treatment response, relapse risk, stem-cell quiescence, and overall survival [14,18–23]. Previous studies have identified biologically and clinically important roles for miRNAs such as miR-29b, miR-181 family members, miR-155, miR-126, miR-22, and miR-125b in regulating DNA methylation, FLT3 signaling, apoptosis, and leukemic stem-cell biology [14,21,22,24–26]. Furthermore, the remarkable stability of circulating miRNAs in blood and serum positions them as attractive minimally invasive biomarkers with strong translational potential [27].

Despite the growing biological evidence supporting miRNAs in AML pathogenesis and prognosis, their integration into clinically applicable prognostic models remains limited. One major challenge is the inherently high-dimensional nature of miRNA datasets, where hundreds of mature miRNAs are typically profiled in relatively small patient cohorts [23,28]. Such settings substantially increase the risk of overfitting and unstable feature selection when conventional statistical or machine-learning approaches are applied. In addition, miRNAs frequently exhibit strong co-expression and regulatory interdependence due to shared transcriptional programs, chromosomal localization, or processing machinery, complicating feature selection strategies based on penalized regression models such as Least Absolute Shrinkage and Selection Operator (LASSO) or Elastic Net [29,30]. Although ensemble-learning and deep-learning approaches can partially address multicollinearity, these models often sacrifice biological interpretability and reproducibility, limiting their translational utility in clinical oncology where compact, explainable biomarker panels are preferred [31].

Several studies have applied machine-learning frameworks to AML prognostication, each with its own dataset, feature space, and evaluation protocol. Tazi *et al.* [32] investigated clinicogenomic risk stratification; Awada *et al.* [33] used genomic signatures for AML subclassification; Mosquera Orgueira *et al.* [34] evaluated gene-expression-based survival prediction; and Eckardt *et al.* [35] studied prediction of complete remission and survival using supervised learning. For miRNA-specific signatures, Garzon *et al.* [20] reported associations between miRNA expression, cytogenetics, and prognosis, and Lim *et al.* [23] developed an expression-based model of event-free survival in pediatric AML. These studies motivate assessment of compact miRNA panels alongside established clinical and molecular predictors. Because their endpoints, cohorts, and validation procedures differ from ours, their reported performance values are not directly comparable with the present results.

Evolutionary learning algorithms provide an approach to high-dimensional biomarker discovery by exploring feature subsets while balancing predictive performance and model complexity [36–38]. The inheritable bi-objective combinatorial genetic algorithm was developed previously for 0/1 combinatorial optimization; its inheritance strategy and orthogonal-array crossover are established methodological contributions [36]. Previous applications include breast cancer diagnosis [38], breast cancer stage classification [39], and survival-associated miRNA discovery in lung adenocarcinoma [40], ovarian cancer [41], and hepatocellular carcinoma [42]. The present study extends this methodological lineage to an AML-specific dataset and candidate signature.

We applied the AML Survival Estimator (AMLS), an evolutionary learning-driven support vector regression framework, to miRNA expression profiles from The Cancer Genome Atlas acute myeloid leukemia (TCGA-LAML) cohort [43] and derived a 28-miRNA candidate panel. To evaluate survival using patient-level censoring information, we linked each profile to patient-level time and event data and evaluated the panel with censoring-aware survival models, a clinical (age/sex) baseline, negative controls, and early-outcome sensitivity analyses. We also report a descriptive comparison of AMLS regression predictions with 10 machine-learning regression approaches, and we annotate the panel’s targets and pathways to explore biological relevance.

## Materials and Methods

### Patient cohort and miRNA expression data

The analysis matrix contained normalized, log2-transformed miRNA expression profiles for 354 features and recorded times for 156 TCGA-LAML patients. Each matrix row was linked to its source sample by exact agreement across all 354 expression values; no patient was assigned by outcome time alone. The linked clinical records provided TCGA patient/sample identifiers, age, sex, vital status, and overall survival time (OS.time). All 156 samples carry TCGA sample-type code 03 (primary blood-derived cancer, peripheral blood) [44]. Of 188 source samples, 13 lacked a numeric OS.time and 19 had a recorded death or follow-up time of 30 d or less; the remaining 156 constituted the primary analysis cohort (Fig. S1; Tables S1 to S3).

Recorded time was defined as the interval between diagnosis and death or last follow-up and converted from days to months by division by 30. The primary cohort contained 96 deaths and 60 right-censored observations. Death times and last-contact times agreed exactly with OS.time for the corresponding event categories. For patient *i*, the survival outcome is (*t_i_*, *δ_i_*), where *t_i_* = min(*T_i_*, *C_i_*), *T_i_* is death time, *C_i_* is censoring time, and *δ_i_* = 1 indicates death and 0 indicates right censoring. In the regression equations, *y_i_* denotes the recorded time, used without an event-dependent loss. Upstream low-expression filtering and normalization steps were not documented in the source records, and no additional filtering was applied. Two early-outcome sensitivity cohorts retained all 162 patients with positive recorded times and all 175 patients with numeric recorded times; the latter included 13 day-zero records as recorded, without time imputation.

### Cross-validation and fixed-panel evaluation

Regression comparators were evaluated using 10-fold cross-validation stratified by quartiles of recorded time (split seed 42). Survival models were evaluated using 3 repeats of event-stratified 10-fold outer cross-validation (seeds 20261014, 20261015, and 20261016), with identical outer partitions for all models. Within each outer training set, hyperparameters were selected by mean Harrell concordance (using predicted risk at 24 mo) across 3 event-stratified inner folds, with standardization fitted separately within every inner and outer training split. All reported survival-model predictions are for held-out outer observations.

The 28-miRNA panel was reconstructed by ranking all 354 features by their appearance counts in the 50 saved AMLS run-specific feature lists and retaining the 28 most frequent features. Panel members appeared in 16 to 49 independent runs; the next-ranked features appeared in 15 runs. Panel discovery used outcome information from the full cohort and preceded survival-model cross-validation. The repeated outer evaluations nested standardization, model fitting, and hyperparameter tuning and therefore estimate performance conditional on the panel. They do not evaluate the complete discovery procedure. Bootstrap intervals and fixed-panel controls have the same scope. Regression analyses summarize the agreement with recorded death or follow-up times. Full-feature elastic-net Cox provides a comparator with coefficient estimation confined to training data. The robust model selected a 28-feature subset overlapping 16 panel members (Table S4).

### AML Survival Estimator

The AMLS combines evolutionary feature selection with support vector regression [36,45]. The archived fitted robust model is a Library for Support Vector Machines (LIBSVM) radial-basis-function nu-Support Vector Regression (ν-SVR) with *C* = 128, *γ* = 0.116865, *ν* = 0.75, and 156 support vectors [46]. Its stored support vectors reproduce the 156 expression rows, in matrix order, after run-specific feature selection and full-cohort min–max scaling (maximum absolute rounding discrepancy < 5.1 × 10^−7^). This correspondence identifies the expression rows and scaling used in the individual fitted model. For standardized inputs *z*, the kernel is

$$K\left(z_{i},z_{j}\right)=\exp\left(-\gamma {\left\|z_{i}-z_{j}\right\|}^{2}\right)$$ (1)

The saved run-specific regression predictor is

$$f\left(z\right)=\sum_{s=1}^{156}\beta_{s}\;K\left(z_{s},z\right)-\rho$$ (2)

where *β* denotes the saved support-vector coefficients and *ρ* the model offset. Equation (3) reconstructs run-specific scaling using the full-cohort minimum and maximum of each selected feature; this differs from the training-only standardization used for survival-model evaluation. The run report documents 10-fold regression evaluation. The model predicts recorded time using a regression objective. A separate Python reimplementation uses a subgradient linear SVR with *C* = 2 and *ε* = 0.5, settings that differ from those of the LIBSVM model.

$$z_{j}=\frac{2\left(x_{j}-a_{\min,j}\right)}{a_{\max,j}-a_{\min,j}}-1$$ (3)

In the Python evolutionary reimplementation, candidate subsets are binary masks *m* in {0,1}*^p^*, with *m_j_* = 1 indicating inclusion of feature *j*. Equation (4) defines a scalar, sparsity-penalized fitness that combines prediction correlation and feature count, rather than an explicitly estimated Pareto front:

Fm=R^innerm−λ·mp (4)

where the implemented correlation is calculated from pooled predictions of an inner 3-fold split. The selected feature count is |*m*|, with *p* = 354, and *λ* = 0.04 controls the sparsity penalty. The implementation uses population size 12, six generations, 2 elite masks, 2-candidate tournament selection, single-point crossover, and independent bit mutation with probability 0.04. With this mutation rate, an expected 354 × 0.04 = 14.16 bits change per child, and no 28-feature cardinality is imposed. Feature supports from this reimplementation (Table S5) are distinct from the AMLS regression predictions.

### Comparator machine-learning models

Ten comparator regression models were evaluated using custom NumPy implementations. Linear approaches included ordinary least squares, ridge (*α* = 10), LASSO (*α* = 0.05), Elastic Net (*α* = 0.05; l1-ratio = 0.5), and a subgradient linear SVR. Nonlinear approaches included a Radial Basis Function (RBF)-kernel regression implementation labeled SVR, *k*-nearest neighbors (*k* = 7; inverse-distance weighting), random forest (40 trees; maximum depth = 8), gradient boosting (50 estimators; learning rate = 0.08), and a single-hidden-layer multilayer perceptron (32 rectified linear units; L2 regularization = 0.001).

Regression scenarios were Full-354 (all expression features) and Sig-28 (the fixed, globally derived panel). The survival-model comparison included ridge-penalized Cox models for age/sex, the 28-miRNA panel, and age/sex plus panel; full-feature elastic-net Cox; and a panel-based random survival forest [47–49]. Cox ridge penalties were 0.1, 1, 10, or 100. Elastic-net Cox used l1-ratio 0.5 and penalties 0.03, 0.1, 0.3, or 1.0. The forest used 200 trees, minimum split size 10, minimum leaf size 5 or 10, and either square-root or all-feature splitting. Every candidate was tuned under the same outer/inner partitions.

### Performance metrics and statistical analysis

Regression summaries included mean absolute error (MAE), root-mean-squared error (RMSE), *R*^2^, Pearson and Spearman correlation, and concordance calculated from recorded times without event indicators. Survival analyses used the verified patient-linked time/event records, censoring-aware objectives, and held-out predictions.

For the recorded times and predictions, the descriptive MAE is

$$\mathrm{MAE}=\frac{1}{n}\sum_{i=1}^{n}\left|y_{i}-{\hat{y}}_{i}\right|$$ (5)

the RMSE

$$\text{RMSE}=\sqrt{\frac{1}{n}\sum_{i=1}^{n}{\left(y_{i}-{\hat{y}}_{i}\right)}^{2}}$$ (6)

the coefficient of determination

$$R^{2}=1-\frac{\sum_{i}^{n}{\left(y_{i}-{\hat{y}}_{i}\right)}^{2}}{\sum_{i}^{n}{\left(y_{i}-\bar{y}\right)}^{2}}$$ (7)

and the Pearson product-moment correlation is calculated between recorded times and regression predictions as

$$R_{\text{Pearson}}=\frac{\sum_{i}^{n}\left(y_{i}-\bar{y}\right)\left({\hat{y}}_{i}-\bar{\hat{y}}\right)}{\sqrt{\sum_{i}^{n}{\left(y_{i}-\bar{y}\right)}^{2}}\cdot \sqrt{\sum_{i}^{n}{\left({\hat{y}}_{i}-\bar{\hat{y}}\right)}^{2}}}$$ (8)

Survival predictions were evaluated with Harrell concordance, Uno concordance restricted to 36 months, cumulative/dynamic area under the curve (AUC) and inverse probability of censoring weighting (IPCW) Brier scores at 12, 24, and 36 months, and integrated Brier score (IBS) over 6 to 36 months on a half-month grid [47,50,51]. Discrimination used predicted death risk at 24 months. Survival curves were averaged across the 3 outer repeats before pooled evaluation; per-repeat results are also provided. Evaluation follow-up was administratively truncated just beyond 36 months for IPCW support, while model fitting retained all recorded times. The censoring distribution for pooled metrics was estimated by Kaplan–Meier on the analysis cohort. Conditional 95% intervals and the paired difference in concordance used 1,000 patient bootstrap samples holding the out-of-fold predictions and panel fixed. Regression fold intervals and Wilcoxon comparisons are reported descriptively. With 10 nonzero pairs, the minimum attainable exact 2-sided signed-rank *P* value is 1/512 (approximately 0.001953).

### miRNA target and pathway annotations

Target annotations in Table S6 were compiled from an offline catalogue attributed to miRTarBase, TargetScan, and miRDB [52–54]. The catalogue distinguishes experimental-support labels from predictions. Feature labels include precursor/locus identifiers, such as hsa-miR-125b-2 and hsa-miR-30c-1, and target interpretation depends on the corresponding mature miRNA species.

The combined catalogue comprises 181 unique target labels and 247 miRNA–target edges. Target and pathway summaries describe the recorded annotations, with pathway-tag frequencies calculated as the number of panel features carrying each tag in Table 1.

**Table 1.** Composition of the 28-miRNA panel. Rows give appearance counts across 50 evolutionary runs, catalogued target counts by evidence category, and qualitative pathway annotations.

Rank	miRNA	Selection frequency (evolutionary runs)	Catalogued experimental targets (*n*)	Predicted targets (*n*)	Key pathways
1	hsa-miR-877	49	5	7	Cell cycle; apoptosis; MAPK signaling; PI3K-AKT signaling
2	hsa-miR-191	41	6	7	AML pathway; hematopoietic cell lineage; cell cycle; TGF-beta signaling
3	hsa-miR-129-1	37	5	5	p53 signaling; cell cycle; apoptosis
4	hsa-miR-323	33	2	6	PI3K-AKT signaling; chemokine signaling; cell cycle
5	hsa-miR-511-2	33	3	4	Innate immune response; Toll-like receptor signaling; AML pathway
6	hsa-miR-874	30	5	5	JAK-STAT signaling; cell cycle; apoptosis; AML pathway
7	hsa-miR-30c-1	29	5	6	Apoptosis; HIF-1 signaling; AML pathway
8	hsa-miR-135b	29	6	5	Wnt signaling; Hippo signaling; PI3K-AKT signaling
9	hsa-miR-545	29	3	5	Cell cycle; VEGF signaling; apoptosis
10	hsa-miR-625	28	4	5	MAPK signaling; PI3K-AKT signaling; TGF-beta signaling
11	hsa-miR-1537	28	0	5	Epigenetic regulation; hematopoietic differentiation
12	hsa-miR-362	24	4	4	NF-kB signaling; AML pathway; cell cycle
13	hsa-miR-1308	24	0	4	p53 signaling; apoptosis
14	hsa-miR-148a	23	7	5	DNA methylation; AML pathway; PI3K-AKT signaling; apoptosis
15	hsa-miR-452	22	5	5	TGF-beta signaling; MAPK signaling; cell cycle
16	hsa-miR-579	21	4	4	MAPK signaling; RAS-RAF signaling; cell cycle
17	hsa-miR-29c	21	9	6	DNA methylation; apoptosis; AML pathway; TGF-beta signaling
18	hsa-miR-125b-2	20	8	5	p53 signaling; apoptosis; AML pathway; hematopoietic cell lineage
19	hsa-miR-194-1	20	5	5	TGF-beta signaling; PI3K-AKT signaling; cell cycle
20	hsa-miR-550-1	20	0	5	Cell cycle; TGF-beta signaling
21	hsa-miR-1229	20	1	5	p53 signaling; apoptosis; PI3K-AKT signaling
22	hsa-miR-639	19	1	5	Chemokine signaling; JAK-STAT signaling
23	hsa-miR-15b	19	7	4	Cell cycle; apoptosis; p53 signaling; AML pathway
24	hsa-miR-627	18	2	5	Cell cycle; JAK-STAT signaling; AML pathway
25	hsa-miR-1538	18	0	4	p53 signaling; cell cycle
26	hsa-miR-10b	17	6	5	Cell migration; PI3K-AKT signaling; TGF-beta signaling; AML pathway
27	hsa-miR-188	16	3	5	Cell cycle; PI3K-AKT signaling
28	hsa-miR-548d-2	16	0	5	Hematopoietic differentiation; MAPK signaling; AML pathway

AML, acute myeloid leukemia

### Survival stratification analysis

Exploratory risk groups were defined by tertiles of 24-month predicted death risk from first-repeat outer-held-out panel-Cox predictions, with 52 patients per group. The tertiles summarize model-predicted risk. Kaplan–Meier curves include censor marks and pointwise 95% intervals. We report group events, median survival, survival at 24 months, overall and pairwise log-rank tests with Benjamini–Hochberg correction of the 3 pairwise tests [55], and age/sex-adjusted Cox hazard ratios with 95% intervals. Rank-transformed Schoenfeld-residual tests assessed proportional hazards.

Robustness analyses repeated model fitting and inner tuning for 100 joint permutations of time/event pairs and 50 independently sampled 28-feature panels, using the first-repeat outer design. The controls used the fixed panel and the permutation *P* value is panel conditioned. Early-outcome sensitivities used one event-stratified 10-fold outer/3-fold inner evaluation of the 3 ridge-Cox models in the 162 positive-time and 175 numeric-time cohorts. At 24 months, calibration compared mean predicted survival with within-tertile Kaplan–Meier survival and pointwise intervals.

### Statistical and computational environment

Survival-model evaluation used Python 3.12.14 with NumPy 2.5.4, pandas 2.3.3, SciPy 1.18.1, scikit-learn 1.9.1, scikit-survival 0.28.0, lifelines 0.30.3, and Matplotlib 3.11.2; the exact executable environment is recorded in the analysis archive [47].

## Results

### Cohort characteristics and miRNA feature landscape

The primary cohort included 156 unique TCGA-LAML patients, with 96 deaths (61.5%) and 60 censored observations (38.5%). Median age was 57 years (interquartile range, 43.75 to 65.25; range 18 to 88), and 83 patients were male and 73 were female. All specimens were primary peripheral-blood samples. Kaplan–Meier median overall survival was 19.23 months, and reverse Kaplan–Meier median follow-up was 40.63 months. Survival at 12, 24, and 36 months was 64.7%, 44.6%, and 31.9%, respectively. Median recorded death or follow-up time was 13.1 months (interquartile range, 7.1 to 27.4), which is distinct from median overall survival (Fig. 1; Table S1).

**Fig. 1.**
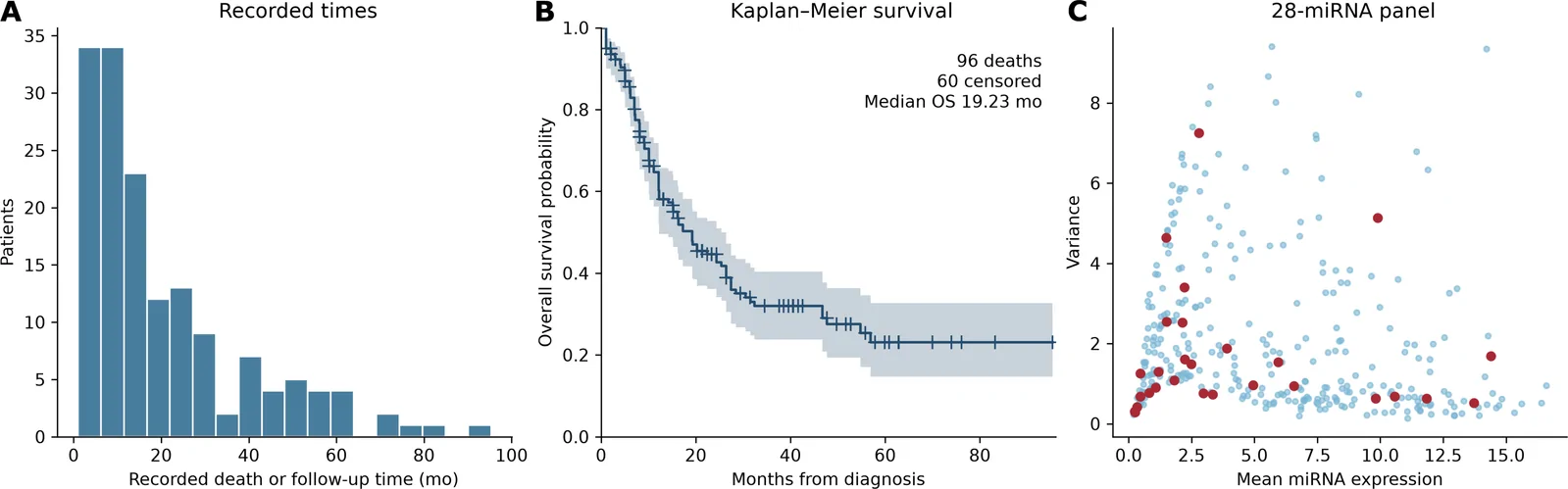
The Cancer Genome Atlas acute myeloid leukemia cohort and expression overview (*n* = 156; *p* = 354). (A) Recorded death or follow-up times. (B) Kaplan–Meier overall survival with censor marks and pointwise 95% confidence intervals (96 deaths; 60 censored; median 19.23 mo). OS, overall survival. (C) Mean–variance expression display highlighting the 28-microRNA (miRNA) panel.

Expression variability across the 354 miRNA features is summarized in the mean–variance display. The 28 panel features were located among the higher-variance features (Fig. 1).

### Descriptive comparison of AMLS regression predictions with comparator models

The AMLS regression prediction series yielded a pooled Pearson *R* of 0.860 and MAE of 7.49 months against recorded time, with pooled unadjusted concordance of 0.788; the corresponding fold means were *R* = 0.858, MAE = 7.53 months, and concordance = 0.791 (Table 2; Fig. 2). The robust model documents a cross-validation MAE of 7.539 months and squared correlation of 0.7344; it is one fitted run and is distinct from the aggregate prediction series.

**Table 2.** Descriptive recorded-time regression metrics. Comparator models use Sig-28 inputs; AML Survival Estimator (AMLS) values summarize the regression prediction series.

Model	Feature set	MAE (mo)	RMSE (mo)	Pearson *R*	Spearman *R*	*R* ^2^	Unadjusted concordance
LASSO	Sig-28	11.94 (10.55–13.34)	15.23 (13.34–17.13)	0.632 (0.530–0.734)	0.613 (0.520–0.706)	0.250 (0.059–0.441)	0.736 (0.698–0.774)
Elastic Net	Sig-28	11.84 (10.47–13.21)	15.10 (13.20–17.01)	0.635 (0.535–0.734)	0.614 (0.523–0.705)	0.268 (0.089–0.448)	0.739 (0.701–0.777)
Ridge	Sig-28	11.68 (10.33–13.03)	14.91 (12.97–16.85)	0.638 (0.543–0.733)	0.612 (0.524–0.700)	0.295 (0.135–0.456)	0.738 (0.704–0.773)
OLS	Sig-28	11.99 (10.58–13.40)	15.30 (13.43–17.17)	0.632 (0.529–0.735)	0.615 (0.521–0.709)	0.239 (0.041–0.438)	0.737 (0.698–0.777)
Linear SVR	Sig-28	11.30 (9.92–12.69)	14.96 (12.92–17.00)	0.616 (0.526–0.705)	0.606 (0.508–0.705)	0.314 (0.192–0.435)	0.732 (0.689–0.775)
Kernel-SVR (RBF)	Sig-28	11.45 (9.51–13.38)	15.14 (12.18–18.11)	0.637 (0.568–0.707)	0.542 (0.456–0.628)	0.331 (0.270–0.392)	0.692 (0.660–0.723)
KNN (*k* = 7)	Sig-28	13.52 (11.07–15.97)	17.31 (13.97–20.65)	0.360 (0.197–0.524)	0.250 (0.094–0.406)	0.113 (−0.012 to 0.239)	0.586 (0.530–0.643)
Random forest	Sig-28	13.83 (11.54–16.13)	17.23 (13.76–20.71)	0.447 (0.338–0.556)	0.361 (0.235–0.487)	0.141 (0.081–0.201)	0.631 (0.582–0.680)
Gradient boosting	Sig-28	14.38 (12.19–16.57)	18.14 (14.75–21.54)	0.328 (0.213–0.444)	0.318 (0.176–0.459)	0.025 (−0.094 to 0.144)	0.610 (0.558–0.663)
MLP (1 × 32)	Sig-28	11.26 (10.25–12.27)	14.50 (13.06–15.94)	0.641 (0.490–0.792)	0.588 (0.471–0.705)	0.284 (0.065–0.504)	0.723 (0.673–0.773)
AMLS	Sig-28	7.53 (6.69–8.37)	9.98 (8.77–11.18)	0.858 (0.808–0.909)	0.741 (0.694–0.788)	0.701 (0.607–0.794)	0.791 (0.772–0.809)

LASSO, Least Absolute Shrinkage and Selection; SVR, Support Vector Regression; RBF, Radial Basis Function; KNN, *k*-Nearest Neighbors; MLP, Multi-Layer Perceptron; MAE, mean absolute error; RMSE, root-mean-square error; OLS, ordinary least squares.

**Fig. 2.**
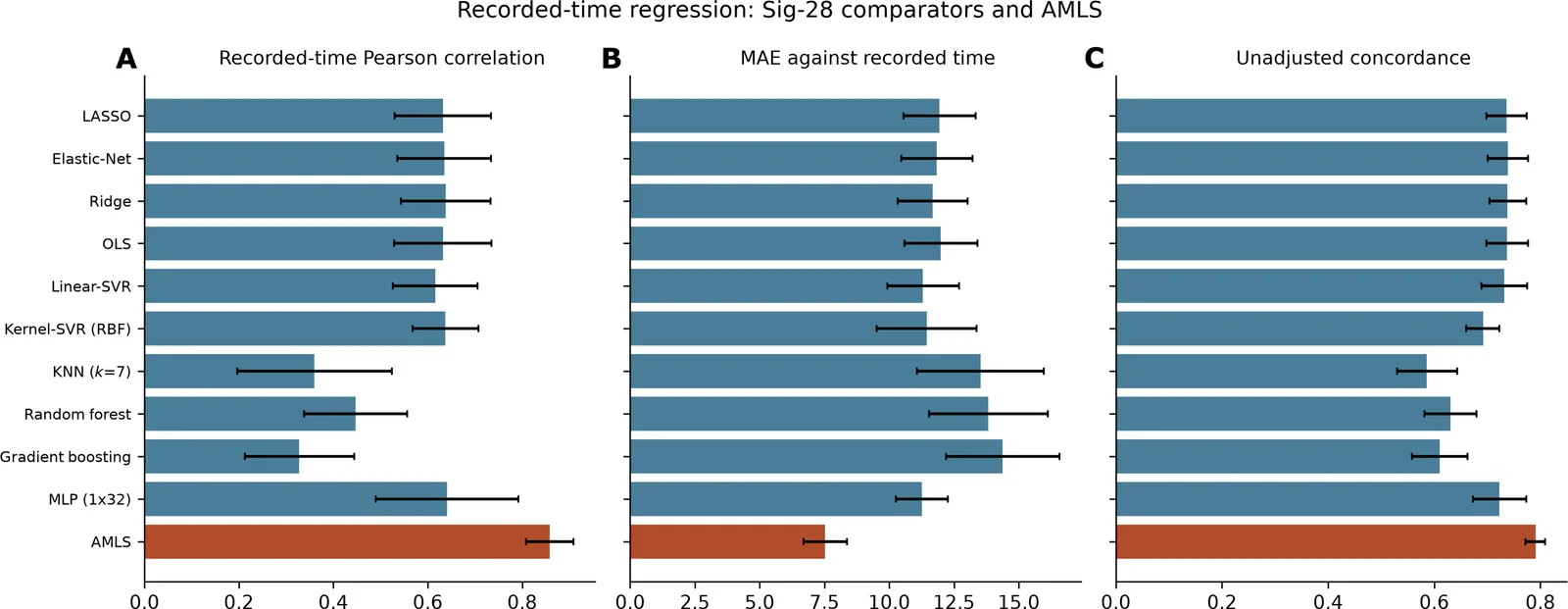
Descriptive recorded-time regression benchmark for the Acute Myeloid Leukemia Survival Estimator (AMLS) and 10 comparator models. Comparator bars use the Sig-28 scenario (Table 2). (A) Fold-mean Pearson *R* with descriptive mean ± 1.96 × SE intervals. OLS, ordinary least squares; LASSO, Least Absolute Shrinkage and Selection Operator; SVR, Support Vector Regression; RBF, Radial Basis Function; KNN, *k*-Nearest Neighbors; MLP, Multi-Layer Perceptron. (B) Mean absolute error (MAE, mo). (C) Concordance calculated from recorded times without event indicators.

With all 354 features as input, fold-mean Pearson *R* was approximately 0.126 for LASSO and 0.177 for Elastic Net under the fixed comparator settings. Per-fold metrics and descriptive intervals are provided in Tables S7 and S8A.

Using the 28-miRNA panel as comparator input yielded fold-mean Pearson *R* = 0.632 for LASSO, 0.635 for Elastic Net, and 0.641 for the Multi-Layer Perceptron (Table 2). These values describe recorded-time regression agreement under the specified inputs and estimator settings. Descriptive Wilcoxon summaries for the recorded-time regression comparisons are provided in Table S8B. Fold-level dispersion is shown in Fig. S2. Table S2 provides the verified 156-patient manifest, including event/censoring indicators, age, sex, specimen identifiers, outer-fold assignment, and held-out panel-Cox survival predictions. All 2,340 model/repeat/patient predictions are supplied in Table S9. Predicted-versus-recorded-time scatter plots and residual displays summarize regression agreement (Fig. 3; Fig. S3). Comparator predictions were compressed in the longest-recorded-time range.

**Fig. 3.**
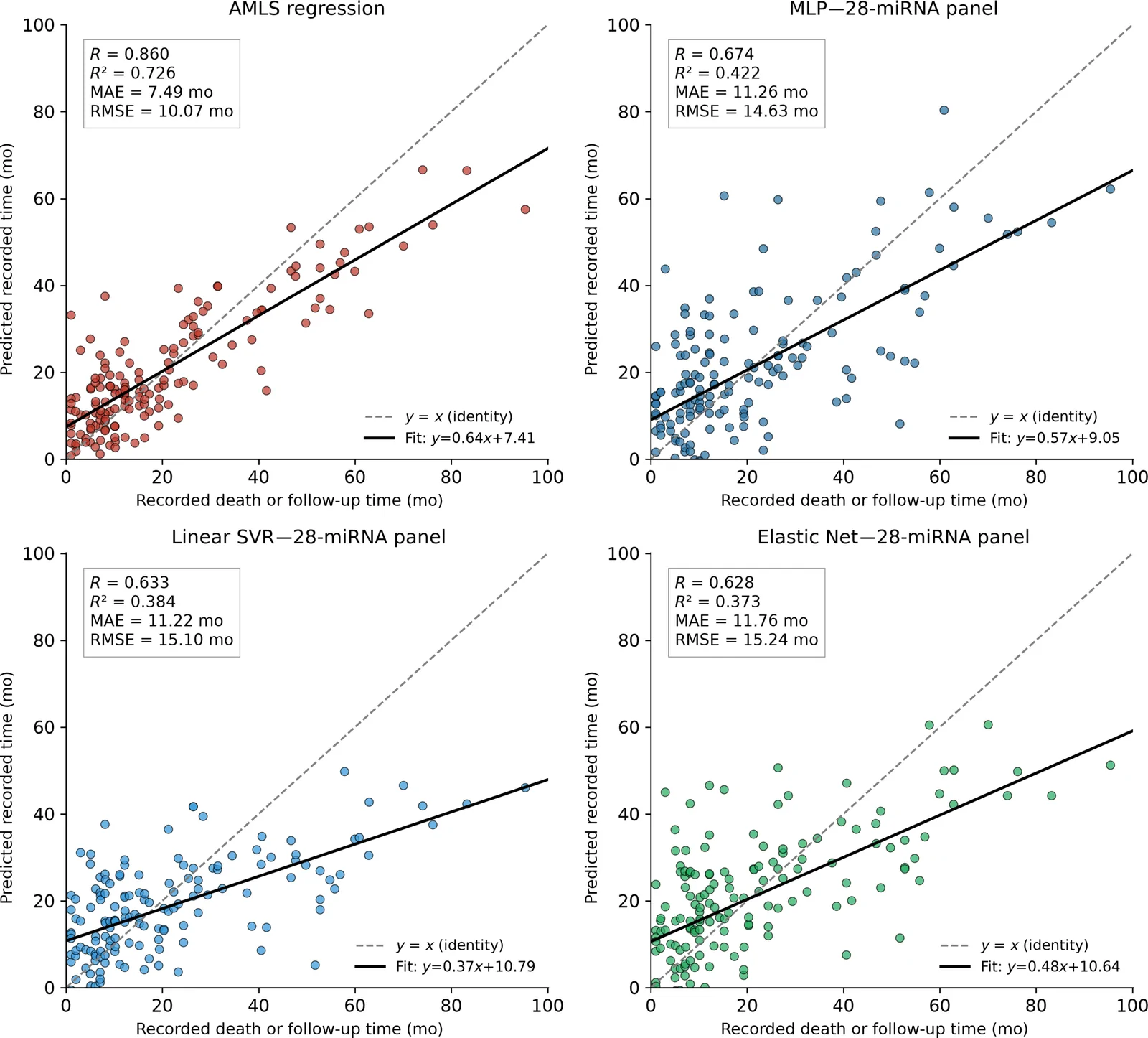
Predicted versus recorded death or follow-up times for the AMLS regression series and 3 comparator models using Sig-28. Dashed line: identity; solid line: fitted regression. RMSE, root-mean-square error.

### Censoring-aware evaluation of the 28-miRNA panel

Across the 3 repeated outer evaluations, the panel-Cox model achieved Harrell *C* = 0.718 (conditional 95% confidence interval [CI], 0.666 to 0.768), Uno *C* at 36 mo = 0.716, 24-month AUC = 0.852, 24-month Brier score = 0.156, and IBS = 0.164. The age/sex model achieved *C* = 0.645 (0.584 to 0.701), AUC24 = 0.685, and IBS = 0.212. Adding age/sex to the panel yielded *C* = 0.730 (0.680 to 0.778), AUC24 = 0.864, and IBS = 0.159. The conditional paired difference in *C* versus age/sex alone was 0.085 (95% CI, 0.027 to 0.147; Table 3; Fig. 4; Tables S9 and S10).

**Table 3.** Censoring-aware survival-model performance in the 156-patient cohort. Predictions are averaged across 3 repeats of outer-held-out evaluation. Patient bootstrap intervals hold predictions and panel membership fixed. Area under the curve (AUC) and Brier scores refer to 24 months; whereas the integrated Brier score (IBS) spans 6–36 months. Full-feature elastic-net Cox uses 354 features; the other molecular models use the 28-miRNA panel.

Model	Harrell *C* (95% CI)	Uno C36	AUC24	Brier24	IBS6–36
Clinical Cox	0.645 (0.584–0.701)	0.639	0.685	0.225	0.212
Panel Cox	0.718 (0.666–0.768)	0.716	0.852	0.156	0.164
Clinical plus panel Cox	0.730 (0.680–0.778)	0.729	0.864	0.151	0.159
Full-feature elastic-net Cox	0.581 (0.524–0.640)	0.587	0.680	0.221	0.217
Panel survival forest	0.684 (0.629–0.737)	0.691	0.804	0.199	0.192

**Fig. 4.**
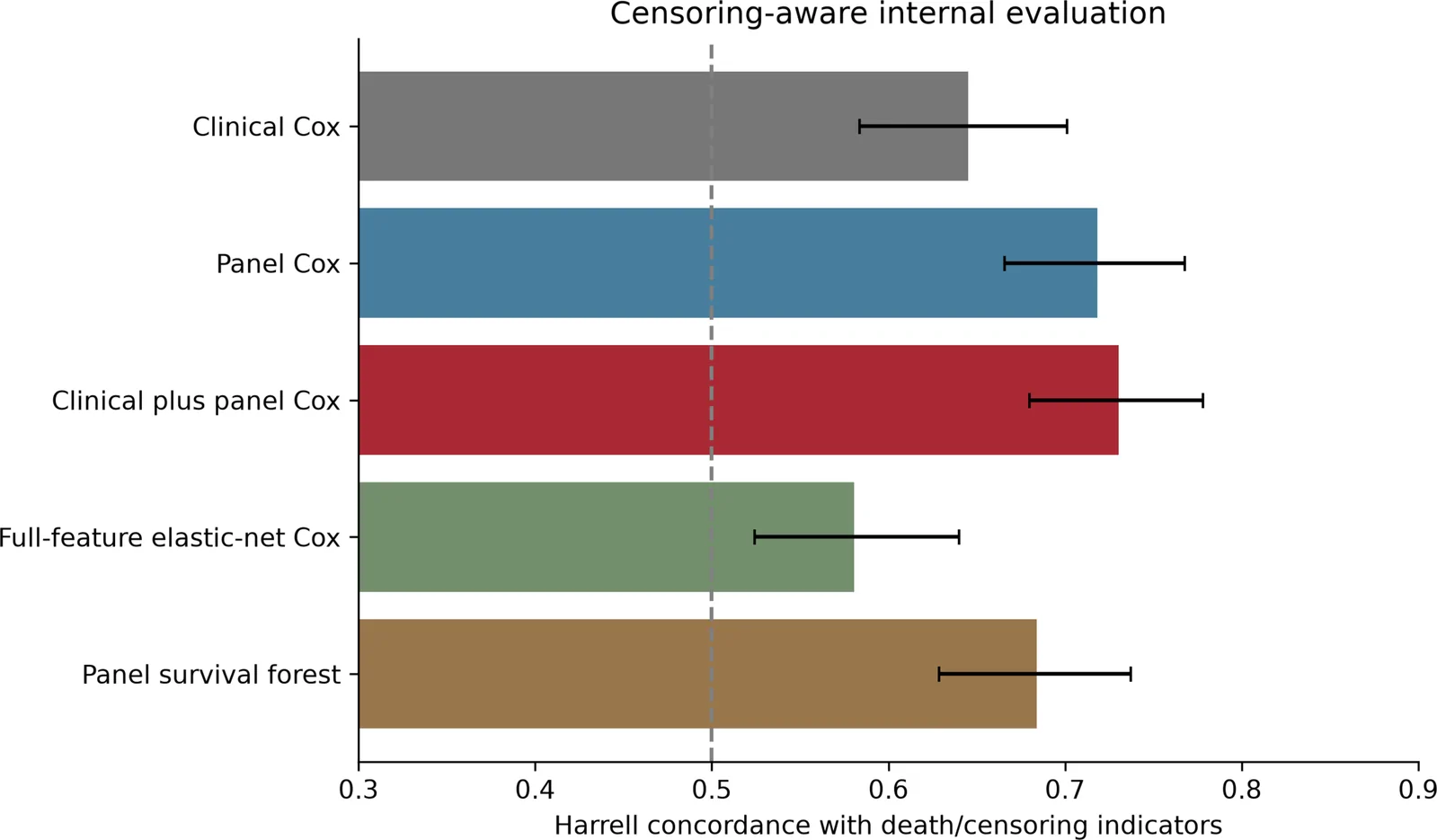
Censoring-aware survival-model performance in the 156-patient cohort. Harrell concordance is calculated from outer-held-out predictions averaged over 3 repeats. Intervals represent 1,000 patient bootstrap samples with predictions and panel membership held fixed.

Full-feature elastic-net Cox achieved *C* = 0.581 (0.524 to 0.640) and IBS = 0.217; the panel survival forest achieved *C* = 0.684 (0.629 to 0.737) and IBS = 0.192. Twenty-four-month calibration was examined by predicted-survival tertiles with Kaplan–Meier intervals (Fig. S4; Table S11). The first-repeat panel-Cox *C* was 0.699. Under 100 joint time/event permutations with refitting and retuning, median *C* was 0.491 (central 95% range 0.404 to 0.598; panel-conditioned *P* = 0.0099). Fifty outcome-independent random 28-feature panels yielded median *C* = 0.599 (range 0.504 to 0.648), with none reaching the selected-panel first-repeat value (Table S12).

Retaining the 6 additional positive-time records produced a 162-patient sensitivity cohort (100 deaths; 62 censored), in which *C* was 0.654 for age/sex, 0.654 for the panel alone, and 0.681 for their combination. Including all 175 numeric-time records, with 13 day-zero times as recorded, yielded 112 deaths and 63 censored observations, with corresponding *C* values of 0.686, 0.704, and 0.726 (Table S13). Panel and age/sex concordance were equal in the 162-patient cohort, while the panel had a numerically higher *C* in the 175-patient cohort. These results demonstrate sensitivity to cohort definition.

### Composition and selection frequency of the 28-miRNA panel

The 28-miRNA panel and its appearance counts across 50 evolutionary runs are listed in Table 1. The retained features are the 28 with the highest appearance frequencies in the saved run lists. The robust selected a 28-feature subset overlapping 16 panel features (Table S4). In the 10 supports from the panel-seeded Python reimplementation, 27 panel members occurred in at least 5 supports; support sizes ranged from 20 to 62 (Figs. S5 and S6).

The panel includes miRNA families with previously reported roles in hematopoiesis, leukemogenesis, apoptosis, and therapeutic resistance, including miR-191, miR-29c, miR-125b, miR-148a, miR-15/16, miR-10b, and miR-30c [25,56–61]. The panel also includes less-characterized candidates for AML-specific functional investigation. The heat map displays interpatient heterogeneity in standardized panel expression, with patients ordered by recorded time (Fig. 5). Co-expression within the panel is shown in Fig. S7. The most frequently selected panel features were hsa-miR-877 (49/50 runs), hsa-miR-191 (41/50), hsa-miR-129-1 (37/50), and hsa-miR-323 (33/50).

**Fig. 5.**
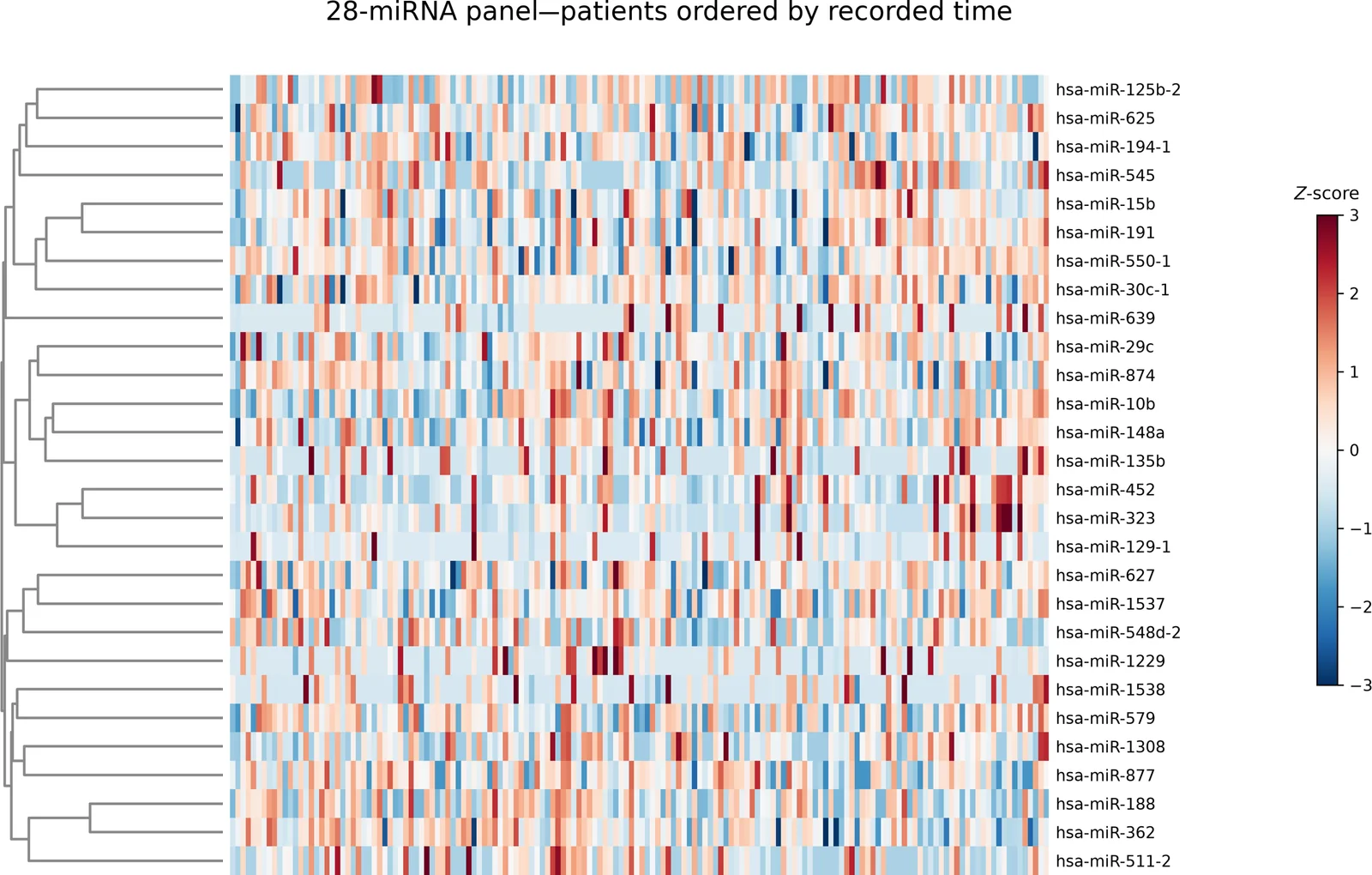
Standardized expression heat map of the 28-miRNA panel across 156 patients ordered by increasing recorded time.

### Exploratory target and pathway annotations

The combined catalogue mapped the panel to 181 unique target labels and 247 miRNA–target annotations, comprising 106 edges labeled experimentally supported and 141 labeled predicted (Fig. 6; Table S6).

**Fig. 6.**
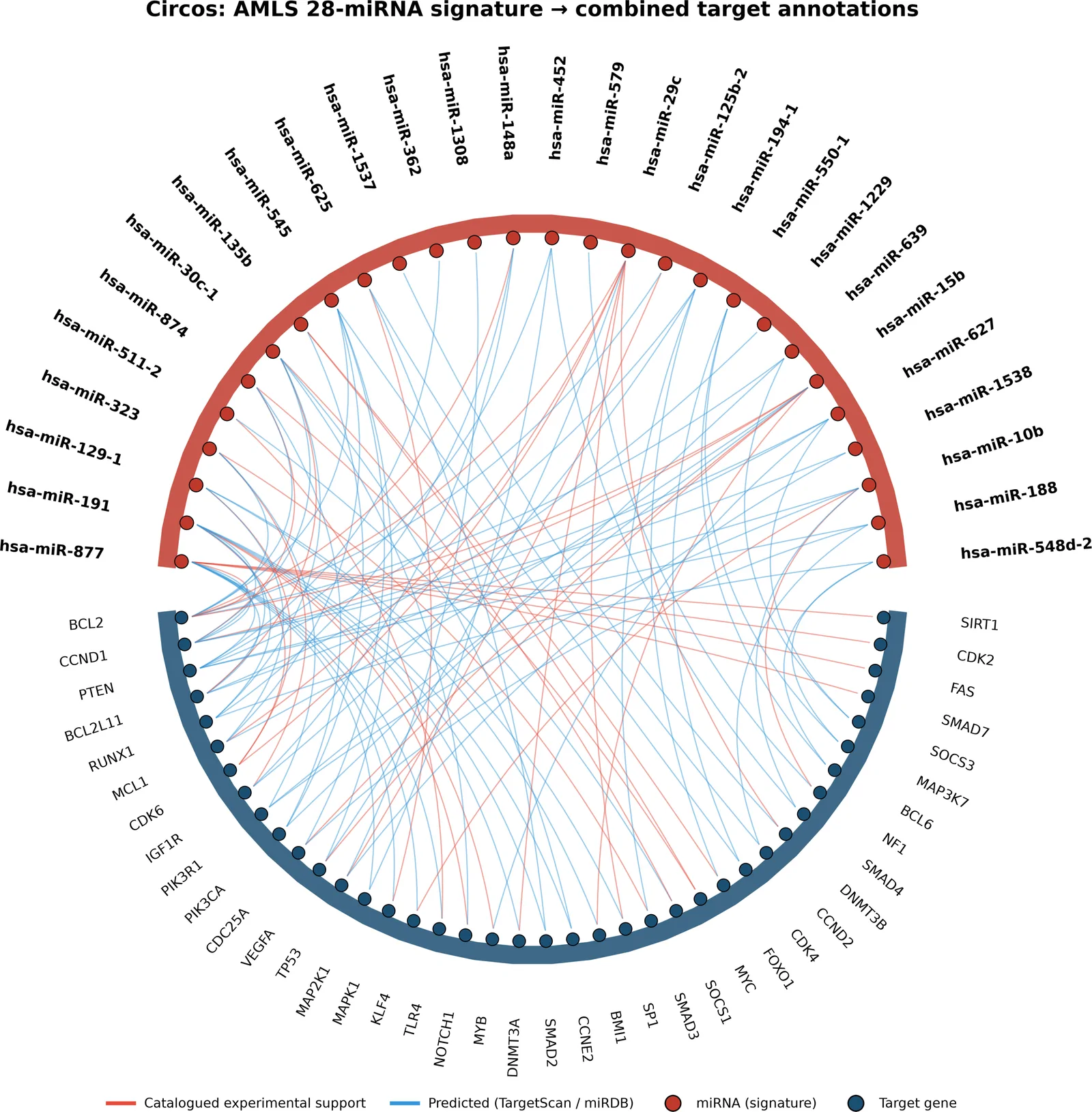
Combined target-annotation network linking the 28-miRNA panel and the 40 most frequently catalogued target labels. Edges show catalogued experimental support (red) or predictions (blue). The complete 247-edge catalogue is provided in Table S6.

Several annotated target genes are established regulators of leukemogenesis, including *DNMT3A*, *DNMT3B*, *BCL2*, *MCL1*, *CDK6*, and *TP53* [1,8,24,62]. Previous studies have investigated the role of miR-29b in DNA methylation in AML, whereas the miR-29c-3p/DNMT3B/LATS1 interaction has been studied in hepatocellular carcinoma [24,63]. The miR-148a/DNMT1 study concerns AML, and the miR-15/16 review concerns chronic lymphocytic leukemia [64,65]. The annotated genes include regulators of BCL2-dependent apoptosis, MCL1-associated resistance, and DNA methylation, providing biological context for pathways relevant to AML therapies [8,62,66]. The network view and detailed Circos displays summarize connections between panel features, catalogued targets, and pathway tags (Figs. S8 and S9).

The annotation catalogues contain 24 Kyoto Encyclopedia of Genes and Genomes/Reactome terms and 18 Gene Ontology terms spanning proliferation, apoptosis, hematopoiesis, and related processes (Tables S14A and B). Catalogued pathway tags include AML, cell cycle, PI3K-AKT signaling, TGF-beta signaling, apoptosis, and hematopoietic cell lineage. Figure 7 summarizes the number of panel features carrying each tag in Table 1. Gene Ontology annotations include myeloid differentiation, apoptotic signaling, DNA methylation, G1/S transition, and protein kinase activity, identifying processes for functional follow-up (Table S14B).

**Fig. 7.**
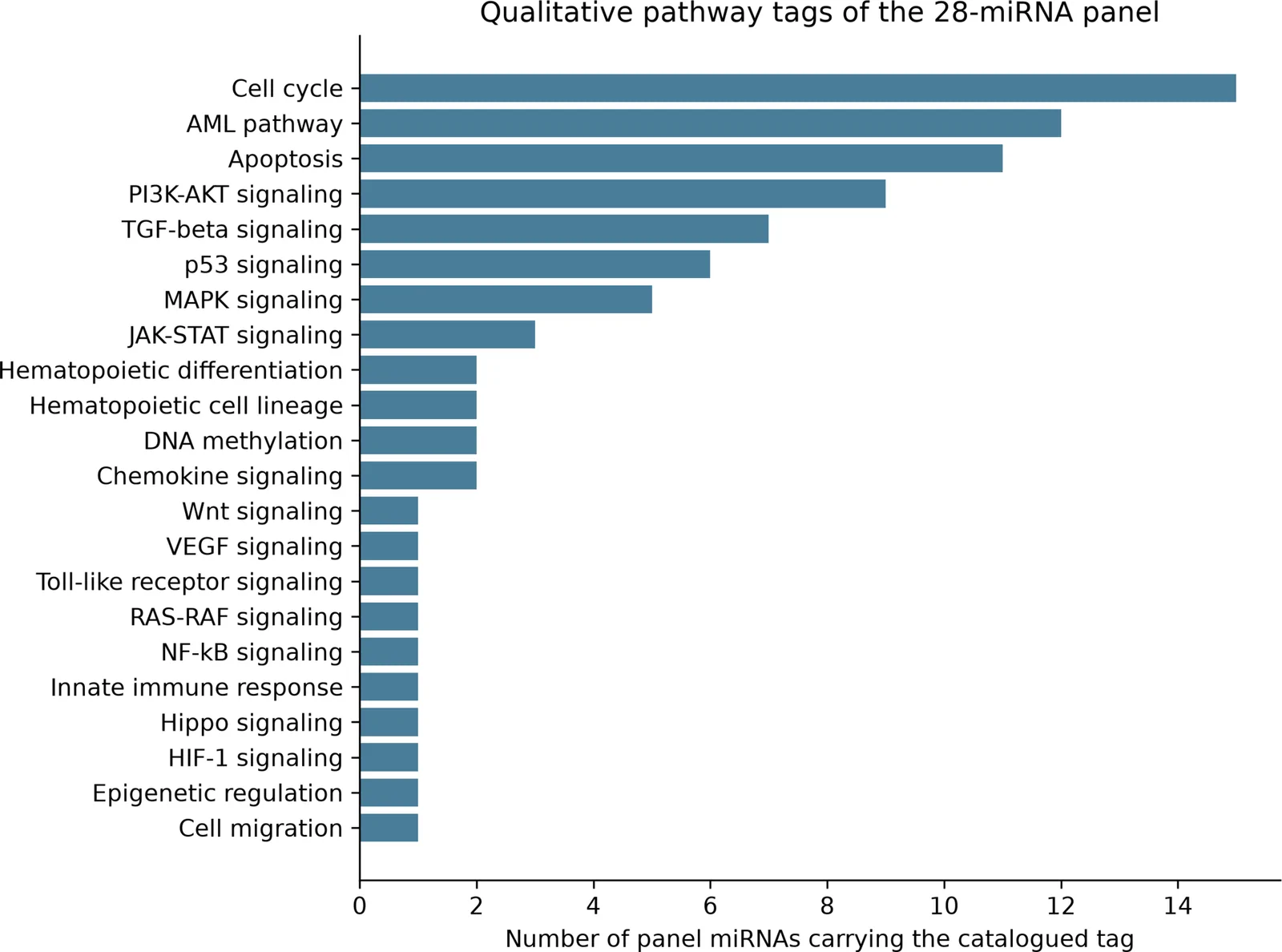
Qualitative pathway-annotation profile of the 28-miRNA panel. Bars count the panel features carrying each pathway tag listed in Table 1.

### Exploratory risk stratification by panel-Cox predicted risk

First-repeat held-out panel-Cox risk at 24 months defined lower, intermediate, and higher tertiles of 52 patients each (Fig. S10; Table S15). Death counts were 24, 29, and 43, with Kaplan–Meier median survival of 46.70, 19.33, and 9.10 months, respectively. Survival at 24 months was 71.5%, 47.2%, and 12.5%.

The overall log-rank *P* value was 2.14 × 10^−10^. Benjamini–Hochberg-adjusted pairwise *P* values were 0.0149 for lower versus intermediate, 1.51 × 10^−9^ for lower versus higher, and 7.10 × 10^−5^ for intermediate versus higher. After age/sex adjustment, hazard ratios relative to the lower group were 1.55 (95% CI, 0.87 to 2.75; *P* = 0.134) for intermediate and 4.49 (2.58 to 7.83; *P* = 1.16 × 10^−7^) for higher risk. Rank-based proportional-hazards tests had *P* ≥0.334 for all 4 terms.

## Discussion

Evolutionary learning identified a 28-miRNA candidate panel for AML, and verified patient linkage enabled its evaluation using censoring-aware survival models. The cohort included 96 deaths and 60 censored observations. The panel-Cox model achieved *C* = 0.718 and IBS = 0.164; adding age/sex yielded *C* = 0.730 and IBS = 0.159. The analyses characterize the panel’s internal prognostic performance and support further investigation alongside clinical and molecular predictors.

Biologically, the panel spans miRNA families implicated in AML and candidates with less extensive disease-specific characterization. Reported miR-29b effects on DNA methylation and associations of miR-29c with AML prognosis support investigation of the miR-29 family [24,57]. Studies of miR-125b in megakaryoblastic leukemia and miR-148a in AML provide additional disease-related context [25,58,65]. Studies of miR-15/16-mediated BCL2 regulation in chronic lymphocytic leukemia and miR-135b in other cancers motivates mechanistic follow-up in AML [59,64,67]. The less-characterized features hsa-miR-877, hsa-miR-129-1, hsa-miR-323, hsa-miR-511-2, hsa-miR-1537, hsa-miR-1308, hsa-miR-550-1, and hsa-miR-548d-2 are further candidates for mature-arm-resolved functional studies.

Methodologically, this study applies the established evolutionary learning framework to an AML-specific biomarker investigation [36]. Repeated survival-model evaluation, a training-only full-feature elastic-net comparator, outcome permutations, and size-matched random panels characterize performance from complementary perspectives. Evaluating evolutionary selection inside each outer training partition with matched estimators would extend the assessment to the complete discovery pipeline.

Our findings complement RNA-based AML prognostic research, including miRNA expression models, immune-related signatures, and competing endogenous RNA analyses [23,68–70]. Earlier applications of the same framework addressed lung, ovarian, and liver-cancer outcomes [38–42]; the present work applies it to a distinct AML cohort and endpoint. Its contribution is an AML-specific candidate panel with documented patient linkage, censoring-aware evaluation, and biological annotation.

Compact miRNA panels may be amenable to targeted assays, and prior analytical work supports studying miRNAs in several specimen types [27,71]. Translation of the cellular peripheral-blood profiles analyzed here to plasma or extracellular-vesicle assays requires separate analytical and biological assessment. Prospective studies can test a locked panel alongside established AML clinical and molecular risk models and examine its potential role in prognostic assessment.

The study has important limitations. The panel was discovered using full-cohort outcome information in a small TCGA-LAML cohort, so the internal estimates and controls may be optimistic. Independent external validation and a fully training-only discovery evaluation remain necessary. Clinical comparisons in the reported analyses used age and sex; incremental value beyond cytogenetic, molecular, treatment, or transplantation variables was not assessed. The 156-patient development matrix excluded recorded outcomes at or before 30 d, and panel performance was attenuated in the 162-patient positive-time sensitivity cohort. The findings therefore establish an exploratory candidate panel, not clinical utility, algorithmic superiority, or mechanistic confirmation.

## Future Directions

Priority next steps include cross-validated evolutionary feature discovery and evaluation, independent validation using compatible miRNA measurements, and comparison with established AML clinical and molecular predictors. Mature-arm-resolved target verification and functional experiments would test candidate mechanisms, and any plasma or extracellular-vesicle application would require separate analytical and biological validation because those assays differ from the cellular peripheral-blood profiles analyzed here. Studies of AML clonal heterogeneity, treatment-associated m6A alterations, and gene-pair ratios in multiple myeloma illustrate complementary molecular approaches [72–74].

## Conclusion

Evolutionary learning identified a compact 28-miRNA candidate panel for AML prognostic research. Patient-level time/event linkage enabled censoring-aware model evaluation, comparison with an age/sex baseline, and complementary sensitivity and control analyses. The panel-Cox and combined models achieved internal concordance of 0.718 and 0.730, respectively. These findings provide a defined panel and quantitative basis for independent evaluation alongside established AML risk models.

## Ethical Approval

This study used de-identified data from The Cancer Genome Atlas (TCGA-LAML), which was conducted under informed consent and Institutional Review Board approval at each contributing institution. No additional ethical approval was required for this secondary analysis.

## Data Availability

TCGA-LAML data are accessible through the Genomic Data Commons (https://portal.gdc.cancer.gov). The supplementary workbook supplies exact sample/patient identifiers, event/censoring variables, cohort flow, the 28-miRNA panel, survival-model fold assignments, hyperparameters, and held-out survival predictions.

## References

[1] Döhner H, Wei AH, Appelbaum FR, Craddock C, DiNardo CD, Dombret H, Ebert BL, Fenaux P, Godley LA, Hasserjian RP, et al. Diagnosis and management of AML in adults: 2022 recommendations from an international expert panel on behalf of the ELN. Blood. 2022;140(12):1345–1377. 10.1182/blood.2022016867 35797463

[2] Shimony S, Stahl M, Stone RM. Acute myeloid leukemia: 2023 update on diagnosis, risk-stratification, and management. Am J Hematol. 2023;98(3):502–526. 10.1002/ajh.26822 36594187

[3] El Chaer F, Bewersdorf JP, Stahl M, Zeidan AM. The global epidemiology of acute myeloid leukaemia. Nat Rev Clin Oncol. 2026;23(2):107–120. 10.1038/s41571-025-01099-7 41350446

[4] DiNardo CD, Erba HP, Freeman SD, Wei AH. Acute myeloid leukaemia. Lancet. 2023;401(10393):2073–2086. 10.1016/S0140-6736(23)00108-3 37068505

[5] Issa GC, Aldoss I, DiPersio J, Cuglievan B, Stone R, Arellano M, Thirman MJ, Patel MR, Dickens DS, Shenoy S, et al. The menin inhibitor revumenib in KMT2A-rearranged or NPM1-mutant leukaemia. Nature. 2023;615(7954):920–924. 10.1038/s41586-023-05812-3 PMC10060155 36922593

[6] Kantarjian H, Chifotides HT, Haddad FG, Jabbour E, Jain N, Kadia T, Ravandi F, Welch MA, Wierda W, Freireich EJ. The care and cure of the leukemias in 2026. Am J Hematol. 2026;101(4):807–831. 10.1002/ajh.70247 41761287

[7] Perner F, Gadrey JY, Armstrong SA, Kühn MWM. Targeting the Menin–KMT2A interaction in leukemia: Lessons learned and future directions. Int J Cancer. 2026;158(2):342–356. 10.1002/ijc.35332 PMC12307729 39887730

[8] DiNardo CD, Jonas BA, Pullarkat V, Thirman MJ, Garcia JS, Wei AH, Konopleva M, Döhner H, Letai A, Fenaux P, et al. Azacitidine and venetoclax in previously untreated acute myeloid leukemia. N Engl J Med. 2020;383(7):617–629. 10.1056/NEJMoa2012971 32786187

[9] Papaemmanuil E, Gerstung M, Bullinger L, Gaidzik VI, Paschka P, Roberts ND, Potter NE, Heuser M, Thol F, Bolli N, et al. Genomic classification and prognosis in acute myeloid leukemia. N Engl J Med. 2016;374(23):2209–2221. 10.1056/NEJMoa1516192 PMC4979995 27276561

[10] Döhner H, Pratz KW, DiNardo CD, Wei AH, Jonas BA, Pullarkat VA, Thirman MJ, Récher C, Schuh AC, Babu S, et al. Genetic risk stratification and outcomes among treatment-naive patients with AML treated with venetoclax and azacitidine. Blood. 2024;144(21):2211–2222. 10.1182/blood.2024024944 PMC11600046 39133921

[11] Bartel DP. Metazoan microRNAs. Cell. 2018;173(1):20–51. 10.1016/j.cell.2018.03.006 PMC6091663 29570994

[12] Marcucci G, Mrózek K, Radmacher MD, Garzon R, Bloomfield CD. The prognostic and functional role of microRNAs in acute myeloid leukemia. Blood. 2011;117(4):1121–1129. 10.1182/blood-2010-09-191312 PMC3056468 21045193

[13] O’Brien J, Hayder H, Zayed Y, Peng C. Overview of microRNA biogenesis, mechanisms of actions, and circulation. Front Endocrinol. 2018;9:402. 10.3389/fendo.2018.00402 PMC6085463 30123182

[14] Lechman ER, Gentner B, Ng SW, Schoof EM, Van Galen P, Kennedy JA, Nucera S, Ciceri F, Kaufmann KB, Takayama N, et al. miR-126 regulates distinct self-renewal outcomes in normal and malignant hematopoietic stem cells. Cancer Cell. 2016;29(2):214–228. 10.1016/j.ccell.2015.12.011 PMC4749543 26832662

[15] Ooi AG, Sahoo D, Adorno M, Wang Y, Weissman IL, Park CY. MicroRNA-125b expands hematopoietic stem cells and enriches for the lymphoid-balanced and lymphoid-biased subsets. Proc Natl Acad Sci USA. 2010;107(50):21505–21510. 10.1073/pnas.1016218107 PMC3003003 21118986

[16] Calin GA, Croce CM. MicroRNA signatures in human cancers. Nat Rev Cancer. 2006;6(11):857–866. 10.1038/nrc1997 17060945

[17] Esteller M. Non-coding RNAs in human disease. Nat Rev Genet. 2011;12(12):861–874. 10.1038/nrg3074 22094949

[18] Mi S, Lu J, Sun M, Li Z, Zhang H, Neilly MB, Wang Y, Qian Z, Jin J, Zhang Y, et al. MicroRNA expression signatures accurately discriminate acute lymphoblastic leukemia from acute myeloid leukemia. Proc Natl Acad Sci USA. 2007;104(50):19971–19976. 10.1073/pnas.0709313104 PMC2148407 18056805

[19] Marcucci G, Maharry KS, Metzeler KH, Volinia S, Wu YZ, Mrózek K, Nicolet D, Kohlschmidt J, Whitman SP, Mendler JH, et al. Clinical role of microRNAs in cytogenetically normal acute myeloid leukemia: MiR-155 upregulation independently identifies high-risk patients. J Clin Oncol. 2013;31(17):2086–2093. 10.1200/JCO.2012.45.6228 PMC3731981 23650424

[20] Garzon R, Volinia S, Liu CG, Fernandez-Cymering C, Palumbo T, Pichiorri F, Fabbri M, Coombes K, Alder H, Nakamura T, et al. MicroRNA signatures associated with cytogenetics and prognosis in acute myeloid leukemia. Blood. 2008;111(6):3183–3189. 10.1182/blood-2007-07-098749 PMC2265455 18187662

[21] Schwind S, Maharry K, Radmacher MD, Mrózek K, Holland KB, Margeson D, Whitman SP, Hickey C, Becker H, Metzeler KH, et al. Prognostic significance of expression of a single microRNA, miR-181a, in cytogenetically normal acute myeloid leukemia cancer and leukemia group B study. J Clin Oncol. 2010;28(36):5257–5264. 10.1200/JCO.2010.29.2953 PMC3018359 21079133

[22] Khalife J, Radomska HS, Santhanam R, Huang X, Neviani P, Saultz J, Wang H, Wu YZ, Alachkar H, Anghelina M, et al. Pharmacological targeting of miR-155 via the NEDD8-activating enzyme inhibitor MLN4924 (Pevonedistat) in FLT3-ITD acute myeloid leukemia. Leukemia. 2015;29(10):1981–1992. 10.1038/leu.2015.106 PMC4868182 25971362

[23] Lim EL, Trinh DL, Ries RE, Wang J, Gerbing RB, Ma Y, Topham J, Hughes M, Pleasance E, Mungall AJ, et al. MicroRNA expression-based model indicates event-free survival in pediatric acute myeloid leukemia. J Clin Oncol. 2017;35(35):3964–3977. 10.1200/JCO.2017.74.7451 PMC5721230 29068783

[24] Garzon R, Liu S, Fabbri M, Liu Z, Heaphy CE, Callegari E, Schwind S, Pang J, Yu J, Muthusamy N, et al. MicroRNA-29b induces global DNA hypomethylation and tumor suppressor gene reexpression in acute myeloid leukemia by targeting directly DNMT3A and 3B and indirectly DNMT1. Blood. 2009;113(25):6411–6418. 10.1182/blood-2008-07-170589 PMC2710934 19211935

[25] Klusmann J-H, Li Z, Böhmer K, Maroz A, Koch ML, Emmrich S, Godinho FJ, Orkin SH, Reinhardt D. miR-125b-2 is a potential oncomiR on human chromosome 21 in megakaryoblastic leukemia. Genes Dev. 2010;24(5):478–490. 10.1101/gad.1856210 PMC2827843 20194440

[26] Jiang X, Hu C, Arnovitz S, Bugno J, Yu M, Zuo Z, Chen P, Huang H, Ulrich B, Gurbuxani S, et al. miR-22 has a potent anti-tumour role with therapeutic potential in acute myeloid leukaemia. Nat Commun. 2016;7(1):11452. 10.1038/ncomms11452 PMC5477496 27116251

[27] Mitchell PS, Parkin RK, Kroh EM, Fritz BR, Wyman SK, Pogosova-Agadjanyan EL, Peterson A, Noteboom J, O’Briant KC, Allen A, et al. Circulating microRNAs as stable blood-based markers for cancer detection. Proc Natl Acad Sci USA. 2008;105(30):10513–10518. 10.1073/pnas.0804549105 PMC2492472 18663219

[28] Bzdok D, Altman N, Krzywinski M. Statistics versus machine learning. Nat Methods. 2018;15(4):233–234. 10.1038/nmeth.4642 PMC6082636 30100822

[29] Tibshirani R. Regression shrinkage and selection via the lasso. J R Stat Soc B Stat Methodol. 1996;58(1):267–288.

[30] Zou H, Hastie T. Regularization and variable selection via the elastic net. J R Stat Soc B Stat Methodol. 2005;67(2):301–320.

[31] Lipton ZC. The mythos of model interpretability in machine learning, the concept of interpretability is both important and slippery. Queue. 2018;16(3):31–57.

[32] Tazi Y, Arango-Ossa JE, Zhou Y, Bernard E, Thomas I, Gilkes A, Freeman S, Pradat Y, Johnson SJ, Hills R, et al. Unified classification and risk-stratification in acute myeloid leukemia. Nat Commun. 2022;13(1):4622. 10.1038/s41467-022-32103-8 PMC9360033 35941135

[33] Awada H, Durmaz A, Gurnari C, Kishtagari A, Meggendorfer M, Kerr CM, Kuzmanovic T, Durrani J, Shreve J, Nagata Y, et al. Machine learning integrates genomic signatures for subclassification beyond primary and secondary acute myeloid leukemia. Blood. 2021;138(19):1885–1895. 10.1182/blood.2020010603 PMC8767789 34075412

[34] Mosquera Orgueira A, Peleteiro Raíndo A, Cid López M, Díaz Arias JÁ, González Pérez MS, Antelo Rodríguez B, Alonso Vence N, Bao Pérez L, Ferreiro Ferro R, Albors Ferreiro M, et al. Personalized survival prediction of patients with acute myeloblastic leukemia using gene expression profiling. Front Oncol. 2021;11: Article 657191. 10.3389/fonc.2021.657191 PMC8040929 33854980

[35] Eckardt JN, Röllig C, Metzeler K, Kramer M, Stasik S, Georgi JA, Heisig P, Spiekermann K, Krug U, Braess J, et al. Prediction of complete remission and survival in acute myeloid leukemia using supervised machine learning. Haematologica. 2023;108(3):690–704. 10.3324/haematol.2021.280027 PMC9973482 35708137

[36] Ho SY, Chen JH, Huang MH. Inheritable genetic algorithm for biobjective 0/1 combinatorial optimization problems and its applications. IEEE Trans Syst Man Cybern B Cybern. 2004;34(1):609–620. 10.1109/tsmcb.2003.817090 15369097

[37] Goldberg DE. Genetic algorithms in search, optimization, and machine learning. Reading (MA): Addison-Wesley; 1989.

[38] Sathipati SY, Tsai MJ, Aimalla N, Moat L, Shukla SK, Allaire P, Hebbring S, Beheshti A, Sharma R, Ho SY. An evolutionary learning-based method for identifying a circulating miRNA signature for breast cancer diagnosis prediction. NAR Genom Bioinform. 2024;6(1):lqae022. 10.1093/nargab/lqae022 PMC10894035 38406797

[39] Yerukala Sathipati S, Ho S-Y. Identifying a miRNA signature for predicting the stage of breast cancer. Sci Rep. 2018;8(1):16138. 10.1038/s41598-018-34604-3 PMC6208346 30382159

[40] Yerukala Sathipati S, Ho S-Y. Identifying the miRNA signature associated with survival time in patients with lung adenocarcinoma using miRNA expression profiles. Sci Rep. 2017;7(1):7507. 10.1038/s41598-017-07739-y PMC5548864 28790336

[41] Sathipati SY, Ho SY. Identification of the miRNA signature associated with survival in patients with ovarian cancer. Aging. 2021;13(9):12660–12690. 10.18632/aging.202940 PMC8148489 33910165

[42] Yerukala Sathipati S, Aimalla N, Tsai MJ, Carter T, Jeong S, Wen Z, Shukla SK, Sharma R, Ho SY. Prognostic microRNA signature for estimating survival in patients with hepatocellular carcinoma. Carcinogenesis. 2023;44(8-9):650–661. 10.1093/carcin/bgad062 37701974

[43] Cancer Genome Atlas Research Network. Genomic and epigenomic landscapes of adult de novo acute myeloid leukemia. N Engl J Med. 2013;368(22):2059–2074. 10.1056/NEJMoa1301689 PMC3767041 23634996

[44] National Cancer Institute. Sample Type Codes. Genomic Data Commons. [accessed 15 Oct 2026] https://gdc.cancer.gov/resources-tcga-users/tcga-code-tables/sample-type-codes

[45] Awad M, Khanna R. Support vector regression. In: *Efficient learning machines*. Berkeley (CA): Apress; 2015. p. 67–80.

[46] Chang CC, Lin CJ. LIBSVM library for support vector machines. ACM Trans Intell Syst Technol. 2011;2(3):1–27.

[47] Pölsterl S. Scikit-survival library for time-to-event analysis built on top of scikit-learn. J Mach Learn Res. 2020;21(212):1–6.

[48] Cox DR. Regression models and life-tables. J R Stat Soc B. 1972;34(2):187–202.

[49] Ishwaran H, Kogalur UB, Blackstone EH, Lauer MS. Random survival forests. Ann Appl Stat. 2008;2:841–860.

[50] Harrell FE, Califf RM, Pryor DB, Lee KL, Rosati RA. Evaluating the yield of medical tests. JAMA. 1982;247(18):2543–2546. 7069920

[51] Uno H, Cai T, Pencina MJ, D’Agostino RB, Wei LJ. On the C-statistics for evaluating overall adequacy of risk prediction procedures with censored survival data. Stat Med. 2011;30(10):1105–1117. 10.1002/sim.4154 PMC3079915 21484848

[52] Cui S, Yu S, Huang HY, Lin YC, Huang Y, Zhang B, Xiao J, Zuo H, Wang J, Li Z, et al. miRTarBase 2025: Updates to the collection of experimentally validated microRNA–target interactions. Nucleic Acids Res. 2025;53(D1):D147–D156. 10.1093/nar/gkae1072 PMC11701613 39578692

[53] Agarwal V, Bell GW, Nam JW, Bartel DP. Predicting effective microRNA target sites in mammalian mRNAs. eLife. 2015;4:e05005. 10.7554/eLife.05005 PMC4532895 26267216

[54] Chen Y, Wang X. miRDBn online database for prediction of functional microRNA targets. Nucleic Acids Res. 2020;48(D1):D127–d131. 10.1093/nar/gkz757 PMC6943051 31504780

[55] Benjamini Y, Hochberg Y. Controlling the false discovery rate practical and powerful approach to multiple testing. J R Stat Soc B. 1995;57(1):289–300.

[56] Szymczyk A, Chocholska S, Radko K, Hus M, Podhorecka M. Pretreatment expression of miR-191a may predict response to the induction chemotherapy based on cytarabine in acute myeloid leukemia patients – A single-center pilotal study. PLOS ONE. 2025;20(6): Article e0324320. 10.1371/journal.pone.0324320 PMC12186893 40554554

[57] Tang LJ, Sun GK, Zhang TJ, Wu DH, Zhou JD, Ma BB, Xu ZJ, Wen XM, Chen Q, Yao DM, et al. Down-regulation of miR-29c is a prognostic biomarker in acute myeloid leukemia and can reduce the sensitivity of leukemic cells to decitabine. Cancer Cell Int. 2019;19(1):177. 10.1186/s12935-019-0894-y PMC6617691 31333331

[58] Ghashghaei M, Le CT, Shaalan H, Escano L, Yue M, Arsalan A, Rouhi A, Nguyen TA, Vu LP. miR-148a-3p and DDX6 functional link promotes survival of myeloid leukemia cells. Blood Adv. 2023;7(15):3846–3861. 10.1182/bloodadvances.2022008123 PMC10393768 36322827

[59] Pekarsky Y, Balatti V, Croce CM. BCL2 and miR-15/16rom gene discovery to treatment. Cell Death Differ. 2018;25(1):21–26. 10.1038/cdd.2017.159 PMC5729525 28984869

[60] Wang CJ, Zou H, Feng GF. MiR-10b regulates the proliferation and apoptosis of pediatric acute myeloid leukemia through targeting HOXD10. Eur Rev Med Pharmacol Sci. 2018;22(21):7371–7378. 10.26355/eurrev_201811_16275 30468483

[61] Katzerke C, Madan V, Gerloff D, Bräuer-Hartmann D, Hartmann JU, Wurm AA, Müller-Tidow C, Schnittger S, Tenen DG, Niederwieser D, et al. Transcription factor C/EBPα-induced microRNA-30c inactivates Notch1 during granulopoiesis and is downregulated in acute myeloid leukemia. Blood. 2013;122(14):2433–2442. 10.1182/blood-2012-12-472183 PMC3790511 23974200

[62] Caenepeel S, Brown SP, Belmontes B, Moody G, Keegan KS, Chui D, Whittington DA, Huang X, Poppe L, Cheng AC, et al. AMG 176, a selective MCL1 inhibitor, is effective in hematologic cancer models alone and in combination with established therapies. Cancer Discov. 2018;8(12):1582–1597. 10.1158/2159-8290.CD-18-0387 30254093

[63] Wu H, Zhang W, Wu Z, Liu Y, Shi Y, Gong J, Shen W, Liu C. miR-29c-3p regulates DNMT3B and LATS1 methylation to inhibit tumor progression in hepatocellular carcinoma. Cell Death Dis. 2019;10(2):48. 10.1038/s41419-018-1281-7 PMC6362005 30718452

[64] Pekarsky Y, Croce CM. Role of miR-15/16 in CLL. Cell Death Differ. 2015;22(1):6–11. 10.1038/cdd.2014.87 PMC4262785 24971479

[65] Wang XX, Zhang H, Li Y. Preliminary study on the role of miR-148a and DNMT1 in the pathogenesis of acute myeloid leukemia. Mol Med Rep. 2019;19(4):2943–2952. 10.3892/mmr.2019.9913 30720097

[66] Stomper J, Rotondo JC, Greve G, Lübbert M. Hypomethylating agents (HMA) for the treatment of acute myeloid leukemia and myelodysplastic syndromes: Mechanisms of resistance and novel HMA-based therapies. Leukemia. 2021;35(7):1873. 10.1038/s41375-021-01218-0 PMC8257497 33958699

[67] Zhao C-C, Jiao Y, Zhang YY, Ning J, Zhang YR, Xu J, Wei W, Kang-Sheng G. Lnc SMAD5-AS1 AS ceRNA inhibit proliferation of diffuse large B cell lymphoma via Wnt/β-catenin pathway by sponging miR-135b-5p to elevate expression of APC. Cell Death Dis. 2019;10(4):252. 10.1038/s41419-019-1479-3 PMC6420660 30874550

[68] Qin L, Li B, Wang S, Tang Y, Fahira A, Kou Y, Li T, Hu Z, Huang Z. Construction of an immune-related prognostic signature and lncRNA–miRNA–mRNA ceRNA network in acute myeloid leukemia. J Leukoc Biol. 2024;116(1):146–165. 10.1093/jleuko/qiae041 38393298

[69] Zhu R, Tao H, Lin W, Tang L, Hu Y. Identification of an immune-related gene signature based on immunogenomic landscape analysis to predict the prognosis of adult acute myeloid leukemia patients. Front Oncol. 2020;10: Article 574939. 10.3389/fonc.2020.574939 PMC7714942 33330048

[70] Xue J, Chen H, Lu J, Zhang H, Geng J, He P, Lu X. Identification of immunity-related lncRNAs and construction of a ceRNA network of potential prognostic biomarkers in acute myeloid leukemia. Front Genet. 2023;14:1203345. 10.3389/fgene.2023.1203345 PMC10301753 37388937

[71] Mestdagh P, Hartmann N, Baeriswyl L, Andreasen D, Bernard N, Chen C, Cheo D, D’andrade P, DeMayo M, Dennis L, et al. Evaluation of quantitative miRNA expression platforms in the microRNA quality control (miRQC) study. Nat Methods. 2014;11(8):809–815. 10.1038/nmeth.3014 24973947

[72] Adhikari S, Eldfors S, Pitkanen E, Vaha-Koskela M, Heckman CA. Whole exome sequencing and single-cell DNA sequencing for assessment of clonal heterogeneity and evolution in acute myeloid leukemia. Comput Struct Biotechnol J. 2025;27:5549–5560. 10.1016/j.csbj.2025.11.066 PMC12731275 41450846

[73] Zhang Z, Zhang L, Li J, Feng R, Li C, Liu Y, Sun G, Xiao F, Zhang C. Comprehensive analysis of m6A methylome alterations after azacytidine plus venetoclax treatment for acute myeloid leukemia by nanopore sequencing. Comput Struct Biotechnol J. 2024;23:1144–1153. 10.1016/j.csbj.2024.02.029 PMC10950754 38510975

[74] Georgiou G, Minadakis G, Karathanasis N, Savva K, Athieniti E, Bourdakou MM, Spyrou GM. Stage-specific gene pair ratios highlight genes and mechanisms related to presymptomatic and symptomatic multiple myeloma. Comput Struct Biotechnol J. 2025;27:3090–3098. 10.1016/j.csbj.2025.07.017 PMC12284557 40703094

