# Supplementary material for: Precision Survival Estimation in Acute Myeloid Leukemia Using Evolutionary Learning-Derived microRNA Signature
Source: Comput Struct Biotechnol J. 2026 Nov 11;35(1):0260. doi: 10.34133/csbj.0260 (PMC13752441; doi:10.34133/csbj.0260)
Supplement: Supplementary file 1 [file csbj.0260.f1.zip › Supplementary Information.docx]

# Supplementary Information

# Supplementary figure legends

**Figure S1.** Cohort flow from 188 source samples to the 156-patient analysis cohort after exclusion of 13 records without numeric survival time and 19 with recorded time at or below 30 days. Sensitivity cohorts comprise 162 patients with positive recorded times and 175 with numeric recorded times.

**Figure S2.** Fold-level distributions of recorded-time Pearson R and MAE for the Sig-28 comparator scenario and AMLS regression summaries.

**Figure S3.** Residual and decile-mean displays of AMLS regression predictions against recorded death or follow-up times.

**Figure S4.** Calibration at 24 months for the age/sex, panel-only, and combined Cox models. Points compare mean three-repeat held-out predicted survival with within-tertile Kaplan–Meier survival. Vertical intervals are pointwise 95% Kaplan–Meier intervals; the diagonal denotes perfect agreement.

**Figure S5.** Selection recurrence in ten feature supports from the panel-seeded Python evolutionary reimplementation. Supports contain 20–62 features; 27 of the 28 panel members occur in at least five supports.

**Figure S6.** Feature appearance counts across 50 evolutionary runs. Orange bars identify the 28 panel members.

**Figure S7.** Pairwise Pearson expression correlations among the 28 panel features.

**Figure S8.** Detailed Circos view of the 28-miRNA panel and the 40 most frequently catalogued target labels. Edge colors distinguish catalogued experimental support from predictions. The complete edge catalogue is provided in Table S6.

**Figure S9.** Circos display linking the 28-miRNA panel to the 14 most frequent qualitative pathway tags in Table 1.

**Figure S10.** Exploratory Kaplan–Meier survival groups defined by tertiles of first-repeat held-out panel-Cox risk at 24 months. Each group contains 52 patients. Curves show censor marks and pointwise 95% confidence intervals.

# Supplementary Table legends

**Table S1.** Characteristics of the 156-patient analysis cohort, including death/censoring counts, age, sex, specimen source, Kaplan–Meier survival, and reverse Kaplan–Meier follow-up.

**Table S2.** Patient-level data in expression-matrix order: TCGA sample/patient identifiers, recorded time, event/censoring indicators, age, sex, specimen source, first-repeat outer-fold assignment, and held-out panel-Cox survival probabilities.

**Table S3.** Cohort-flow records for all 188 source samples, including patient identifiers, numeric-time availability, event status, clinical variables, and analysis-cohort inclusion or exclusion reason.

**Table S4.** Feature membership and model parameters for the individual AMLS robust model, including overlap with the 28-miRNA panel.

**Table S5.** Feature indices in ten supports from the Python evolutionary reimplementation.

**Table S6.** Catalogued miRNA–target annotations comprising 181 unique target labels and 247 interactions.

**Table S7.** Fold-level recorded-time regression metrics by model and scenario.

**Table S8a.** Descriptive fold-mean intervals calculated as mean ± 1.96 × SE across ten fold values.

**Table S8b.** Archived descriptive Wilcoxon summaries for the recorded-time regression comparisons.

**Table S9.** All 2,340 outer-held-out patient/model/repeat predictions, fold seeds, and selected hyperparameters.

**Table S10.** Censoring-aware metrics for all five models, including conditional bootstrap intervals and horizon-specific AUC and Brier scores. Per-repeat results are provided in the analysis archive.

**Table S11**. Twenty-four-month calibration by model-specific predicted-survival tertiles and cohort Kaplan–Meier estimates.

**Table S12**. Panel-conditioned outcome-permutation and size-matched random-panel controls with model refitting and hyperparameter tuning.

**Table S13**. Survival-model metrics in the 162-patient positive-time and 175-patient numeric-time sensitivity cohorts.

**Table S14a.** Qualitative KEGG/Reactome annotation catalogue with term, reported overlap, set size, and catalogue-source labels.

**Table S14b.** Qualitative GO annotation catalogue with term, reported overlap, set size, and ontology labels.

**Table S15.** Exploratory risk-group counts and survival, age/sex-adjusted Cox estimates, BH-adjusted pairwise log-rank tests, and proportional-hazards diagnostics.
