# Supplementary material for: Precision Survival Estimation in Acute Myeloid Leukemia Using Evolutionary Learning-Derived microRNA Signature
Source: Comput Struct Biotechnol J. 2026 Nov 11;35(1):0260. doi: 10.34133/csbj.0260 (PMC13752441; doi:10.34133/csbj.0260)
Supplement: Supplementary file 1 [file csbj.0260.f1.zip › Supplementary_Figures.pdf]

## Supplementary figures

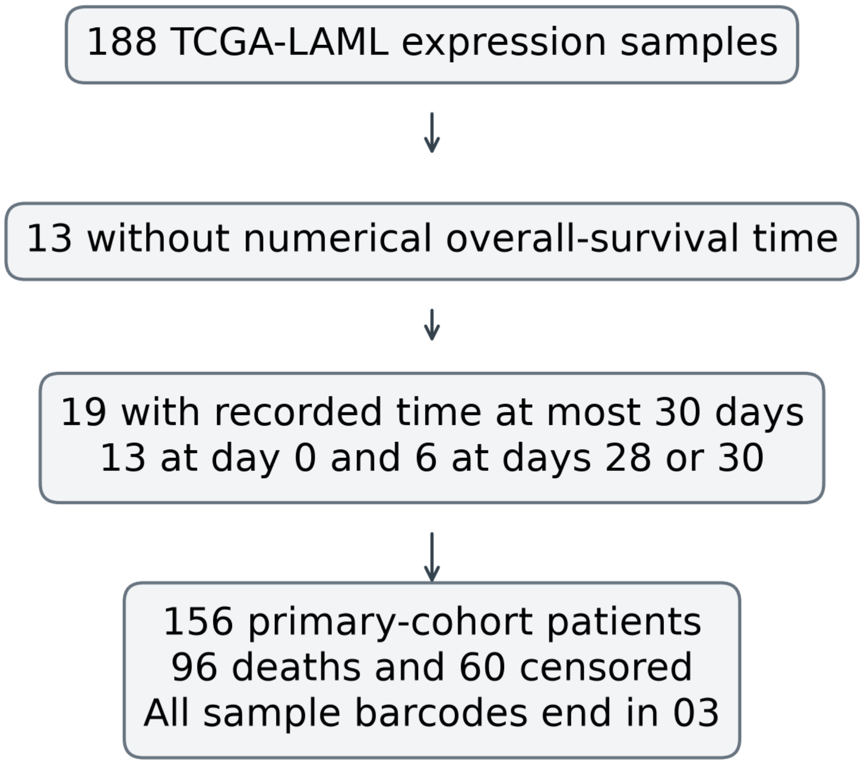


**Supplementary Figure S1.** Cohort flow from 188 source samples to the 156-patient analysis cohort after exclusion of 13 records without numeric survival time and 19 with recorded time at or below 30 days. Sensitivity cohorts comprise 162 patients with positive recorded times and 175 with numeric recorded times.

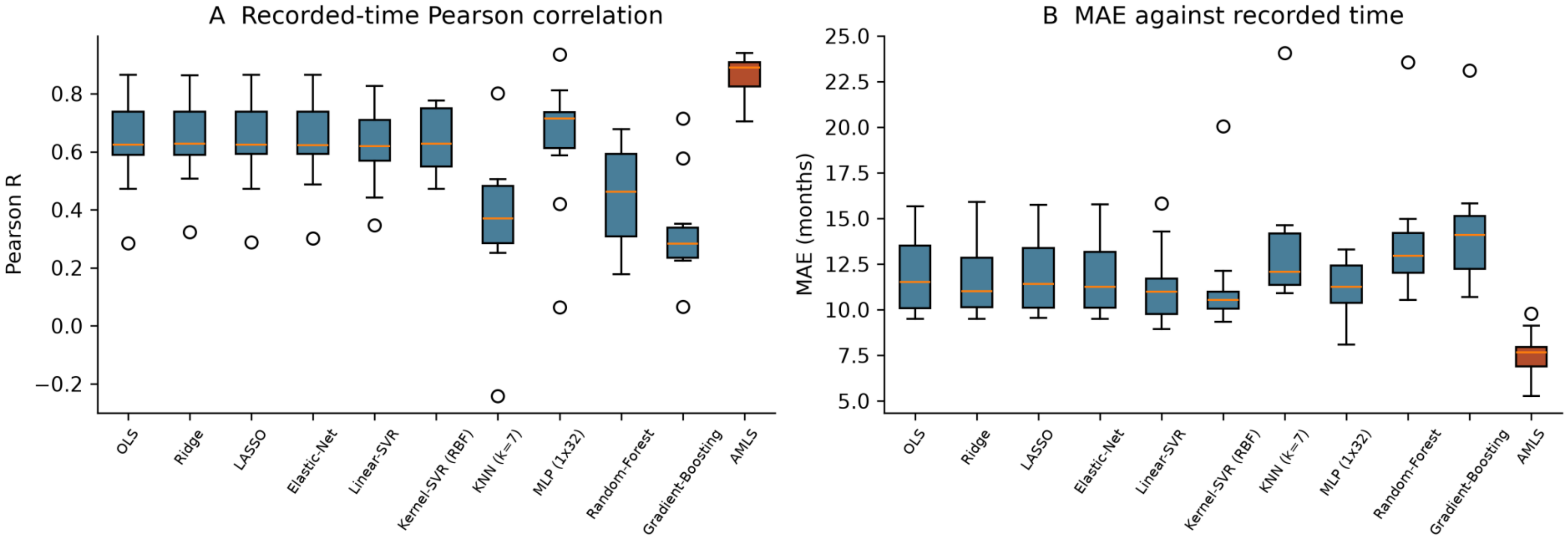


**Supplementary Figure S2.** Fold-level distributions of recorded-time Pearson R and MAE for the Sig-28 comparator scenario and AMLS regression summaries.

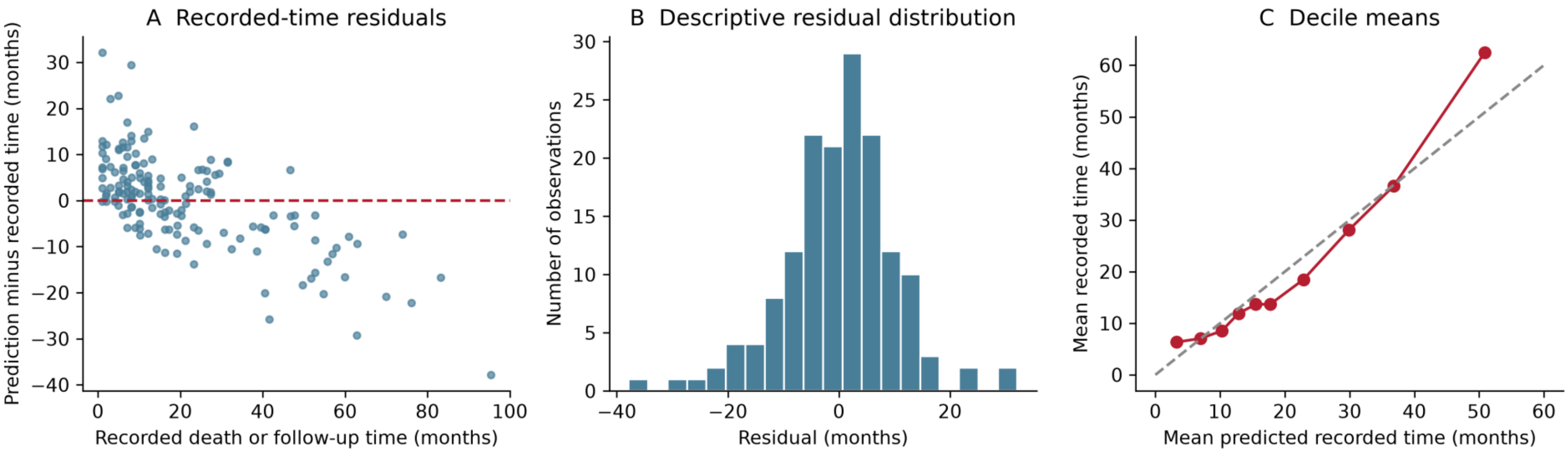


**Supplementary Figure S3.** Residual and decile-mean displays of AMLS regression predictions against recorded death or follow-up times.

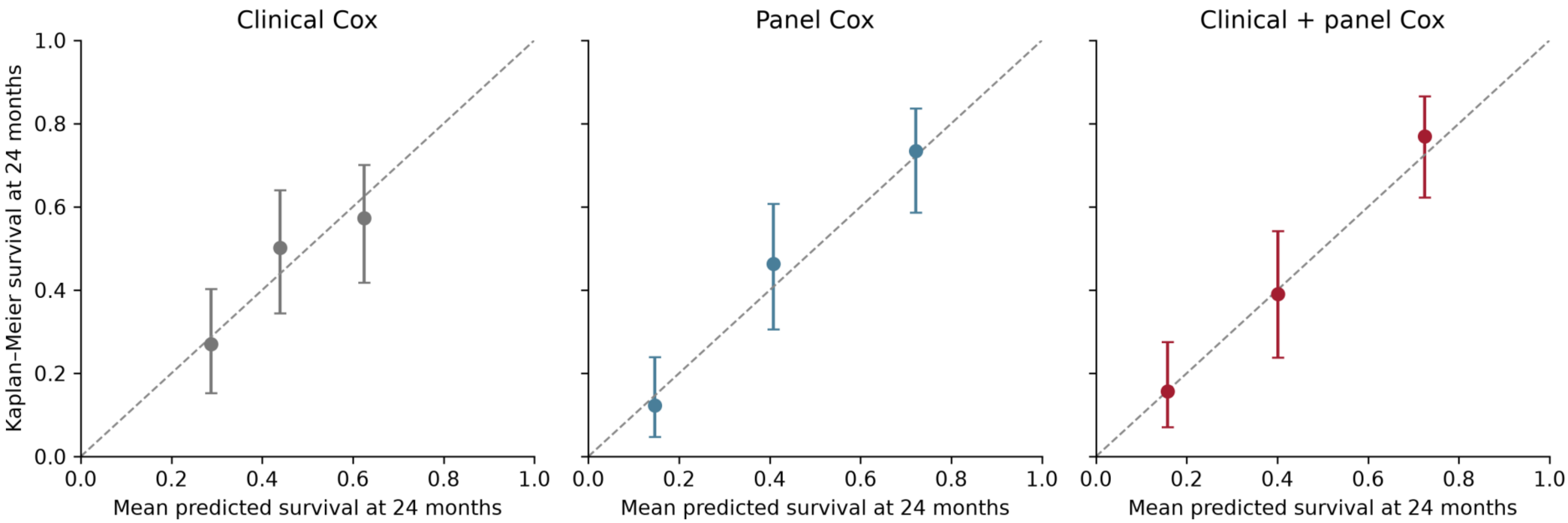


**Supplementary Figure S4.** Calibration at 24 months for the age/sex, panel-only, and combined Cox models. Points compare mean three-repeat held-out predicted survival with within-tertile Kaplan–Meier survival. Vertical intervals are pointwise 95% Kaplan–Meier intervals; the diagonal denotes perfect agreement.

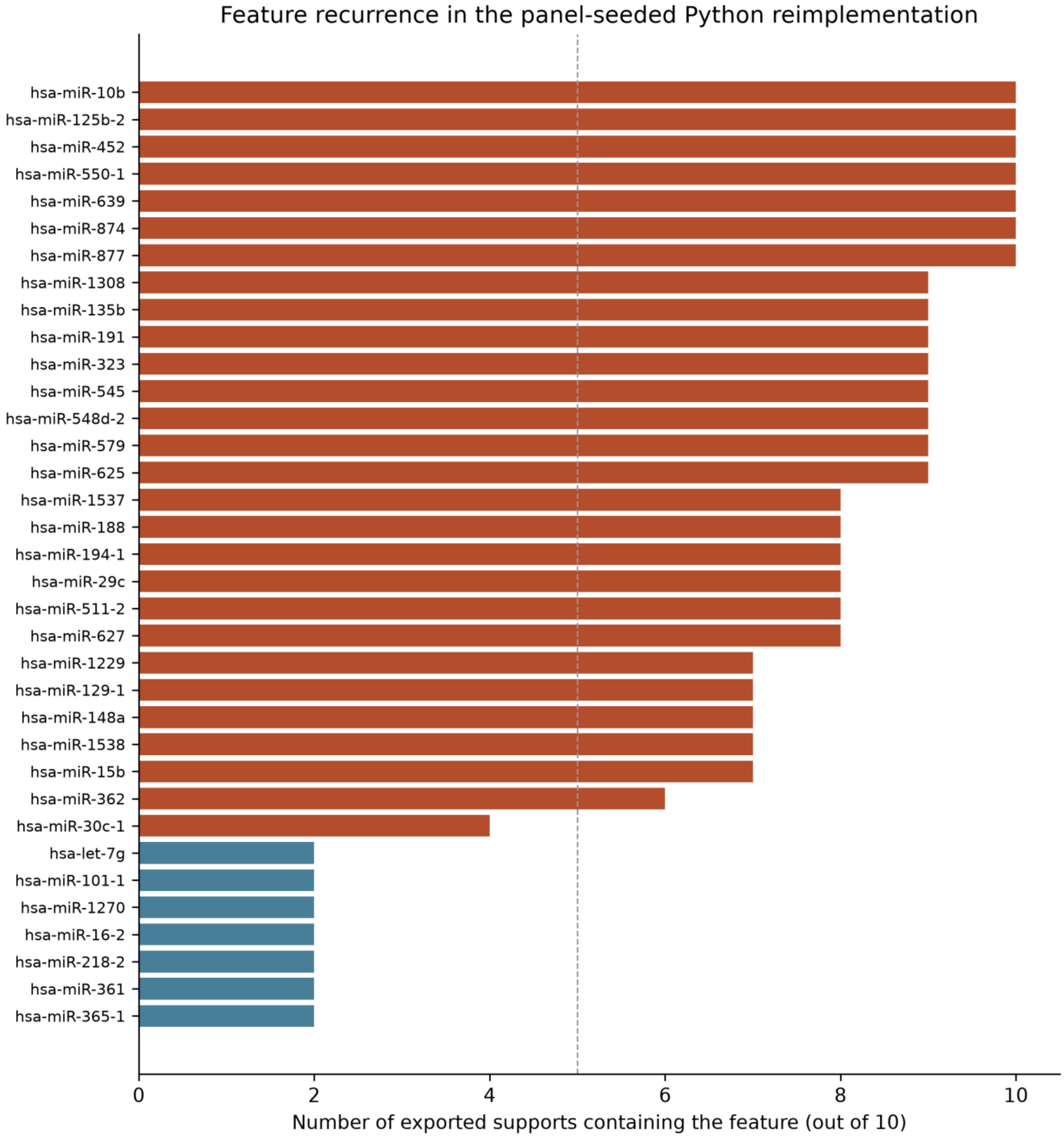


**Supplementary Figure S5.** Selection recurrence in ten feature supports from the panel-seeded Python evolutionary reimplementation. Supports contain 20–62 features; 27 of the 28 panel members occur in at least five supports.

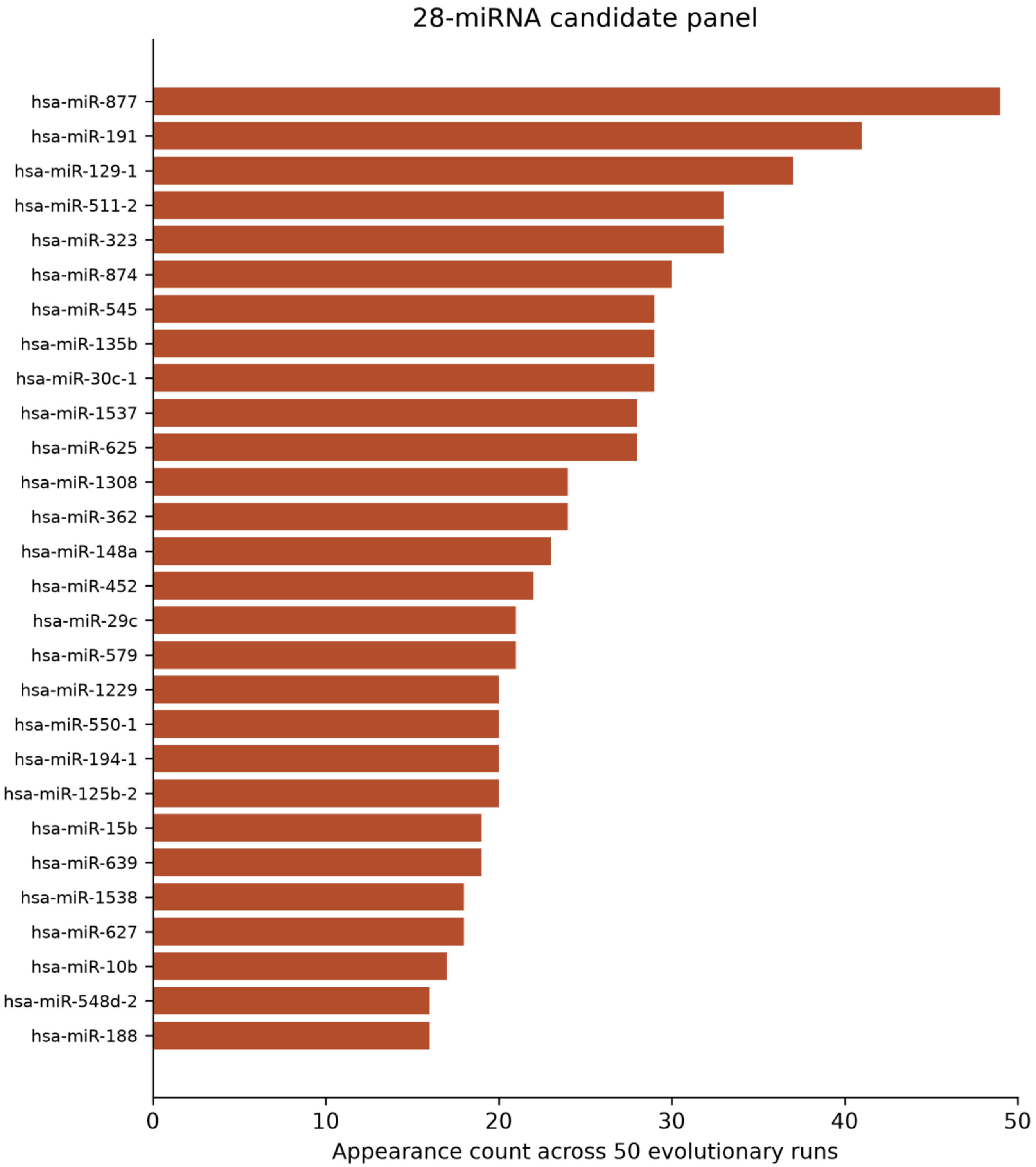


**Supplementary Figure S6.** Feature appearance counts across 50 evolutionary runs. Orange bars identify the 28 panel members.

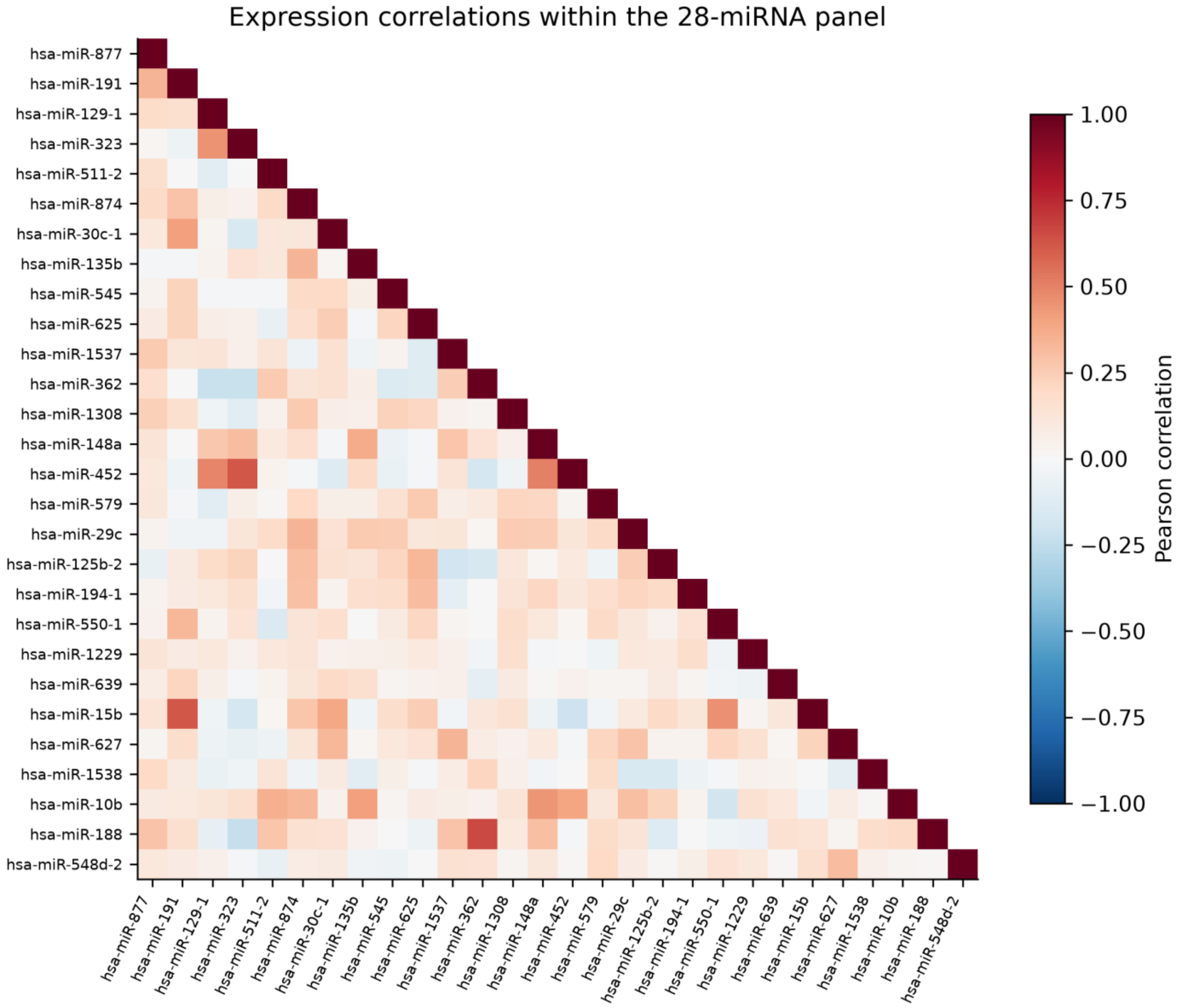


**Supplementary Figure S7.** Pairwise Pearson expression correlations among the 28 panel features.

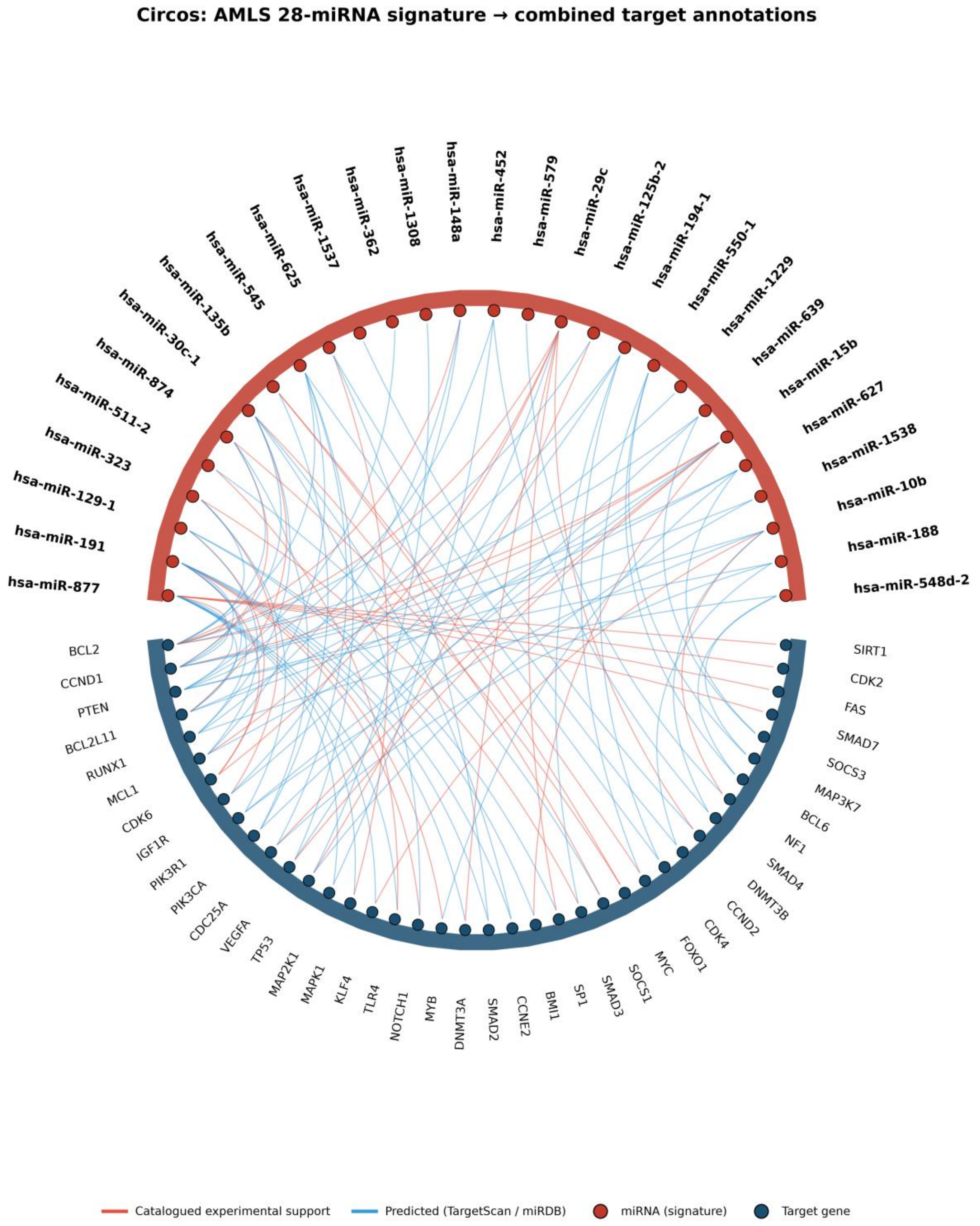


**Supplementary Figure S8.** Detailed Circos view of the 28-miRNA panel and the 40 most frequently catalogued target labels. Edge colors distinguish catalogued experimental support from predictions. The complete edge catalogue is provided in Table S6.

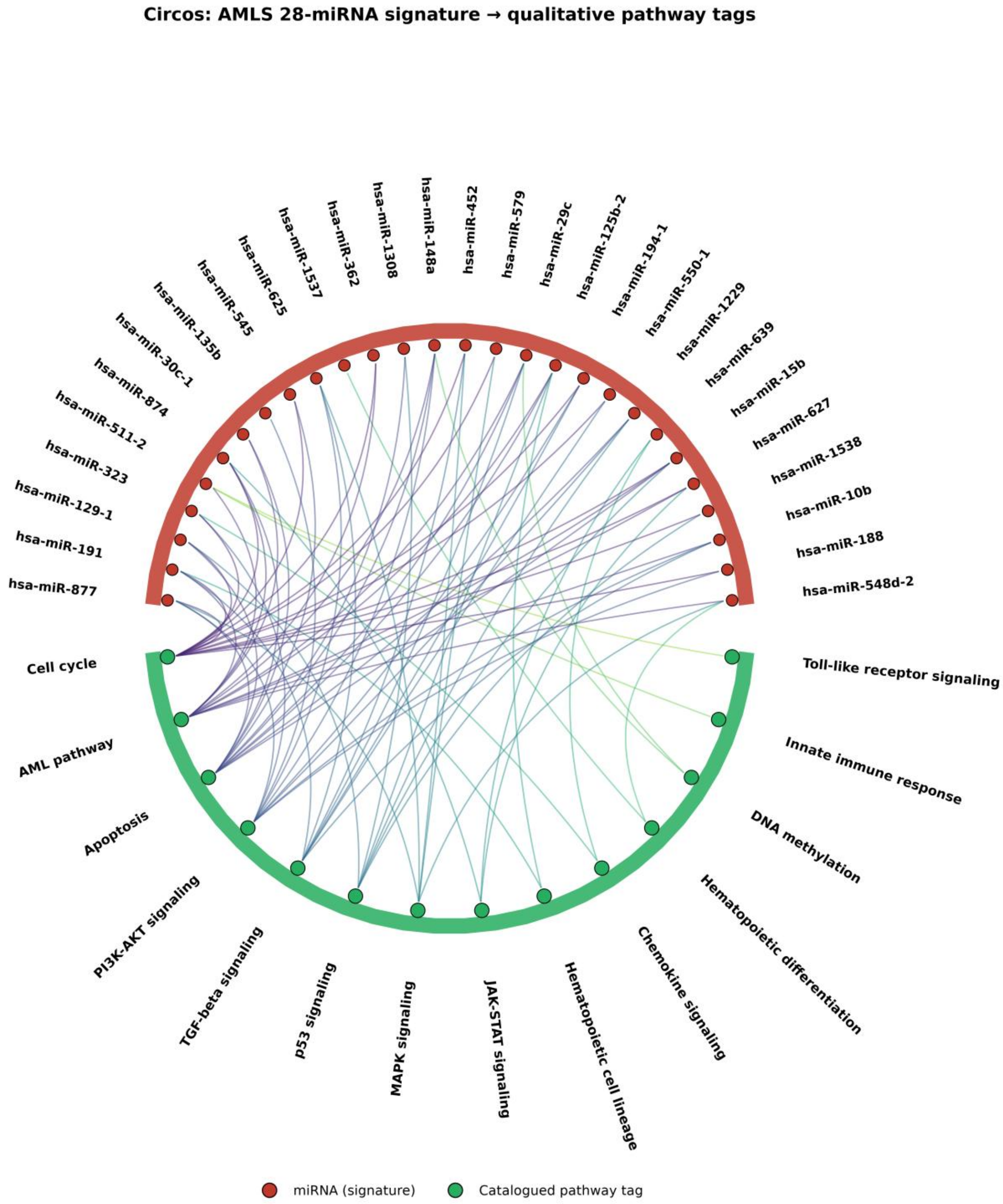


**Supplementary Figure S9.** Circos display linking the 28-miRNA panel to the 14 most frequent qualitative pathway tags in Table 1.

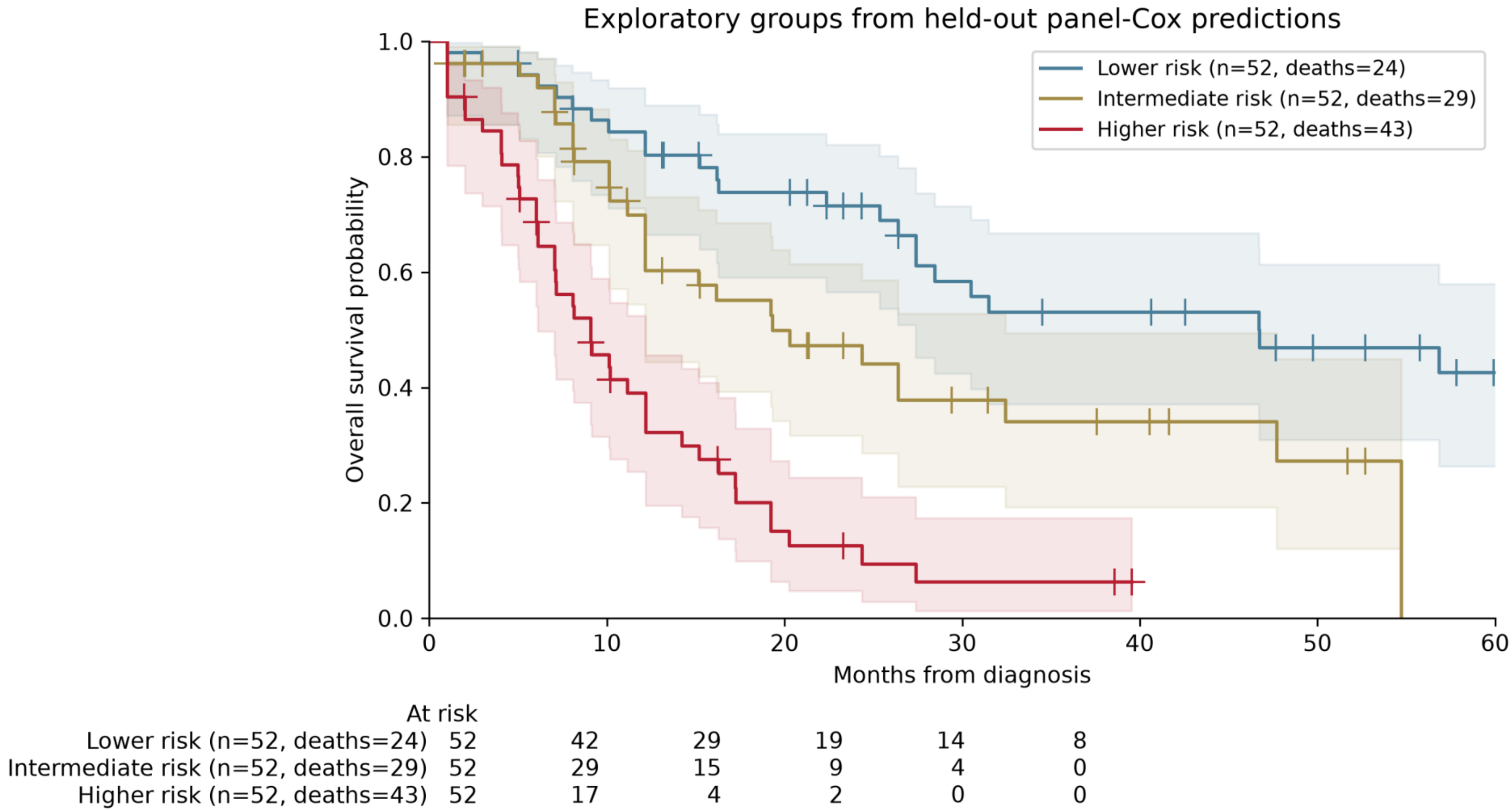


**Supplementary Figure S10.** Exploratory Kaplan–Meier survival groups defined by tertiles of first-repeat held-out panel-Cox risk at 24 months. Each group contains 52 patients. Curves show censor marks and pointwise 95% confidence intervals.
